# Germinal center activity and B cell maturation are associated with protective antibody responses against *Plasmodium* pre-erythrocytic infection

**DOI:** 10.1371/journal.ppat.1010671

**Published:** 2022-07-06

**Authors:** Ganesh Ram R. Visweswaran, Kamalakannan Vijayan, Ramyavardhanee Chandrasekaran, Olesya Trakhimets, Samantha L. Brown, Vladimir Vigdorovich, Ashton Yang, Andrew Raappana, Alex Watson, William Selman, Meghan Zuck, Nicholas Dambrauskas, Alexis Kaushansky, D. Noah Sather

**Affiliations:** 1 Seattle Children’s Research Institute, Seattle, Washington; 2 Department of Pediatrics, University of Washington, Seattle, Washington; 3 Department of Global Health, University of Washington, Seattle, Washington; 4 Brotman Baty Research Institute, Seattle, Washington; 5 Institute for Stem Cell and Regenerative Medicine, Seattle, Washington; Francis Crick Institute, UNITED KINGDOM

## Abstract

Blocking *Plasmodium*, the causative agent of malaria, at the asymptomatic pre-erythrocytic stage would abrogate disease pathology and prevent transmission. However, the lack of well-defined features within vaccine-elicited antibody responses that correlate with protection represents a major roadblock to improving on current generation vaccines. We vaccinated mice (BALB/cJ and C57BL/6J) with *Py* circumsporozoite protein (CSP), the major surface antigen on the sporozoite, and evaluated vaccine-elicited humoral immunity and identified immunological factors associated with protection after mosquito bite challenge. Vaccination achieved 60% sterile protection and otherwise delayed blood stage patency in BALB/cJ mice. In contrast, all C57BL/6J mice were infected similar to controls. Protection was mediated by antibodies and could be passively transferred from immunized BALB/cJ mice into naïve C57BL/6J. Dissection of the underlying immunological features of protection revealed early deficits in antibody titers and polyclonal avidity in C57BL/6J mice. Additionally, *Py*CSP-vaccination in BALB/cJ induced a significantly higher proportion of antigen-specific B-cells and class-switched memory B-cell (MBCs) populations than in C57BL/6J mice. Strikingly, C57BL/6J mice also had markedly fewer CSP-specific germinal center experienced B cells and class-switched MBCs compared to BALB/cJ mice. Analysis of the IgG γ chain repertoires by next generation sequencing in *Py*CSP-specific memory B-cell repertoires also revealed higher somatic hypermutation rates in BALB/cJ mice than in C57BL/6J mice. These findings indicate that the development of protective antibody responses in BALB/cJ mice in response to vaccination with *Py*CSP was associated with increased germinal center activity and somatic mutation compared to C57BL/6J mice, highlighting the key role B cell maturation may have in the development of vaccine-elicited protective antibodies against CSP.

## Author summary

Identifying specific features of vaccine-elicited antibody responses that are associated with protection from malaria infection is a key step toward the development of a safe and effective vaccine. Here we compared antibody and B cell responses in two mouse strains that exhibited a differential ability to generate antibodies that protect from infection challenge. We found that protection was due to the presence of vaccine-elicited antibodies and could be transferred between strains, and that the ability of antibodies to neutralize the parasite was directly linked to the strength (affinity) with which it binds CSP. Thus, we sought to understand if there were differences in the two strains in the process of B cell maturation that leads to generation of high affinity, protective antibody responses after vaccination. Overall, our comparative analysis indicates that germinal center (GC) activity, a key process in B cell maturation, was significantly diminished in the non-protected strain. Further, we observed evidence of higher levels of somatic mutation, which is a result of germinal center activity, in protected mice. Thus, our results indicate that the ability to generate protective antibody responses was linked to enhanced B cell maturation in the protected strain, providing a key clue to the type of responses that should be generated by future vaccines.

## Introduction

Malaria remains a major public health crisis, with more than 241 million cases resulting in more than 627,000 deaths in 2020, concentrated in sub-Saharan Africa and disproportionately affecting pregnant women and children [1,2]. After peaking in 2004, cases have steadily declined for more than a decade, but in recent years cases have plateaued and slightly increased, highlighting the urgent need for new counter measures to achieve eradication [1,2]. Vaccines that prevent infection with *Plasmodium* parasites, the causative agents of malaria, offer the best hope to overcome this plateau and facilitate eradication. While vaccines are in development for all stages of the *Plasmodium* life cycle, the pre-erythrocytic (PE) stage is an attractive target, as stopping the parasite at this asymptomatic stage would prevent infection, subsequent disease, and transmission [3]. RTS,S, the only malaria vaccine with regulatory approval [4–6], and the most clinically advanced whole sporozoite vaccine, *Pf*SPZ [7], both target this stage and result in only partial protection in field trials. Recently, a new PE vaccine candidate, R21, achieved promising results in early clinical field trials [8]. Subunit vaccines, such as RTS,S and R21, induce potent antibody responses against the major surface protein on the sporozoite, the Circumsporozoite Protein (CSP) [5,8]. While whole sporozoite vaccines also elicit anti-CSP antibodies, they also produce antibodies to other *Plasmodium* antigens [9,10].

The mechanisms by which pre-erythrocytic (PE) vaccines prevent malaria infection are yet to be fully characterized, but both antibodies and CD8^+^ T cells have been implicated, depending on the vaccine modality [11–13]. Antibodies have been shown to mediate anti-parasitic activity and are thought to work primarily in the skin and interstitial tissues where they interfere with the sporozoite’s motility and survival [14]. This has been confirmed by studies of monoclonal antibodies (mAbs) targeting CSP, which have been shown experimentally to reduce or prevent *Plasmodium falciparum* infection [14]. The study of anti-CSP mAbs isolated from humans has implicated antibody affinity, epitope specificity, and B cell clonal selection as key factors mediating protective function [15]. Together these studies indicate that antibodies are a key mediator of protection in subunit PE vaccines, and that protective antibodies have inherent features that determine neutralizing capacity. Whereas mAbs have been instrumental to our understanding of antibody-mediated protection from malaria, vaccination with CSP induces complex polyclonal responses consisting of a diversity of antibodies. The key features of vaccine-elicited polyclonal antibody responses that determine protection from infection have yet to be fully defined, but their characterization would be a critical milestone toward the development of an effective CSP-based vaccine.

Here, we aimed to identify the characteristics of protective antibody responses elicited by full-length CSP vaccination using the rodent malaria, *P*. *yoelii*. The *P*. *yoelii* model of malaria infection enabled us to conduct live vaccination and mosquito bite challenges in a wildtype experimental system to dissect the correlates of antibody-mediated protection. To identify features associated with efficacy, we characterized vaccine-elicited B cell responses in two highly diverged mouse strains, BALB/cJ and C57BL/6J, that exhibited differential vaccine-mediated sterilizing immunity. Comparison of immune responses in these two strains enabled us to assess for major differences that may influence protection from infection. We evaluated serum antibody responses, characterized CSP-specific B cell phenotypes, in response to CSP vaccination. We found that vaccine-elicited anti-CSP antibodies alone were able to achieve sterile protection from infection, and that protection was associated with higher magnitude germinal center (GC) responses and somatic mutation of CSP-specific B cells. These findings imply that B cell maturation is a critical determinant in the development of potent sterilizing antibody-mediated immunity against malaria and indicate that vaccine modalities aimed at inducing mature B cell responses will be necessary to achieve sterilizing immunity in the field.

## Results

### Immunization with *Py*CSP elicits anti-parasitic antibodies and sterile protection from *P*. *yoelii* in BALB/cJ, but not C57BL/6J mice

**Fig 1:**
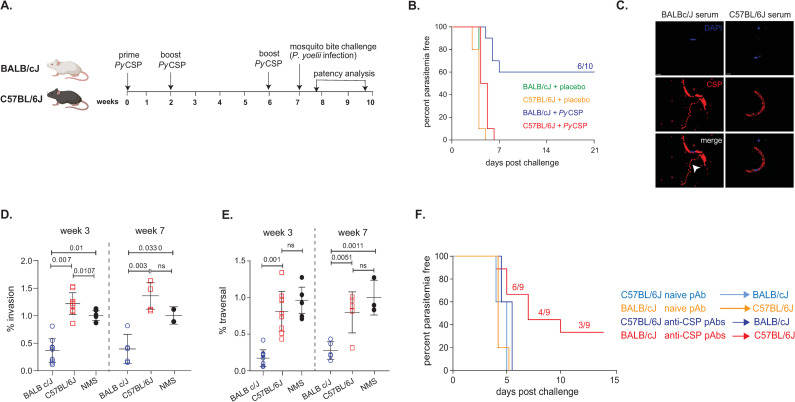
The *Py*CSP-immunization in BALB/cJ and C57BL/6J mice delays blood stage patency and confers protection only in the prior. **A.** Immunization and challenge regimen. **B.** Immunized BALB/cJ and C57BL/6J were challenged with *P*. *yoelii* infected mosquitoes and patency assessed day 3 through day 21. The Kaplan-Meier Survival plots represent percentage parasitemia free mice over time. Data are from 10 mice/condition across 2 independent experiments. 6 out of 10 *PyCSP*-immunized BALB/cJ mice (blue line) remained parasitemia free. **C.** Immunofluorescent images of neutralizing antibody induced CSP shedding in *P*. *yoelii* sporozoites. Sporozoites were incubated with purified serum antibodies, and fluor-coupled mAb 2F6 was used to detect CSP. Nuclei were identified by DAPI staining. Shedding of CSP was induced by BALB/cJ antibodies (white arrow), but not in C57BL/6J serum. (**D** and **E**) Inhibition of sporozoite invasion and traversal. **D**) BALB/cJ immune serum but not C57BL/6J immune serum, significantly reduces sporozoite entry into Hepa1-6 cells. Sporozoites were pre-incubated with serum antibodies 10 mins prior to challenge on Hepa 1–6 cells and entry quantified by flow cytometry. (**E**). BALB/cJ antibodies inhibit fibroblast traversal, whereas C57BL/6J antibodies do not. Antibody-treated sporozoites were exposed to Human Foreskin Fibroblast cells (HFF-1) in the presence of Dextran-FITC for 90 mins, and traversal was quantified by flow cytometry. For (**D**) and (**E**), data represents mean values ± SE from three independent experiments: n = 3. p values were determined by comparing each treatment to untreated using one-way ANOVA for multiple comparisons tests. **F.** Sterile protection from infection in C57BL/6J mice can be achieved by passive administration of purified polyclonal antibodies from *Py*CSP-immunized BALB/cJ mice. Naïve BALB/cJ (n = 5) and C57BL/6J (n = 9) were passively transferred (i.p.) with 90 μg of *Py*CSP antibodies from *Py*CSP-immunized C57BL/6J (blue) and BALB/cJ (red) mice, respectively. Five days post antibody passive transfer, mice were challenged with mosquito-bites from 15 *Py-*infected mosquitoes and the blood stage patency is monitored for 2 weeks. Naïve BALB/cJ (n = 5) and C57BL/6J (n = 5) that were passively transferred with purified polyclonal antibodies from naïve C57BL/6J (light blue) and BALB/cJ (orange) mice, respectively were used as controls. Data analyzed by Two-way ANOVA and p values were obtained by Tukey’s multiple comparison test.

The major strains used to study vaccine efficacy and host pathogen interactions in murine models of malaria infection are BALB/cJ and C57BL/6J [16,17]. In our studies of CSP vaccine-elicited vaccine efficacy, we observed that these two strains exhibited differential levels of protection in a CSP protein subunit immunization, mosquito bite challenge model, where we routinely failed to achieve sterilizing immunity in C57BL/6J mice. To compare efficacy directly, we immunized animals (n = 10 per group) with 20 μg of full length ecto-domain recombinant *Py*CSP in 20% Adjuplex adjuvant at weeks 0, 2 and 6 (Fig 1A). Control groups received PBS-formulated with 20% Adjuplex. Adjuplex is a mixture of Carbopol and nano-liposomes and is known to elicit very high titers of antibodies in mice[18]. At weeks 3 and 7, blood samples were collected to evaluate the antibody responses elicited by the vaccination. At week 7, both mouse strains were subjected to *P*. *yoelii* 17XNL sporozoite infection by mosquito-bite challenge (15 mosquitoes/mouse) and were monitored for three weeks or until blood-stage patency was reached. All the placebo-injected mice in both the BALB/cJ and C57BL/6J control groups developed blood-stage patency in as early as 5 days post mosquito bite challenge, indicating that the strains were equally susceptible to infection (Fig 1B), as has been reported previously [19]. All of the *Py*CSP-vaccinated C57BL/6J mice were unprotected and developed blood-stage patency 6 days post mosquito bite challenge. Surprisingly, in contrast to the C57BL/6J mice, *Py*CSP-immunized BALB/cJ mice exhibited sterile protection from infection (60%). Six BALB/cJ mice were parasite free at the end of the experiment, and the remaining mice (40%) exhibited a significant delay in blood stage patency (7 days) (Figs 1B and S1A).

To study the underlying characteristics of the antibody responses that may mediate sterile protection, we analyzed the anti-parasitic activity of serum polyclonal antibodies (pAbs) *in vitro* and *in vivo*. Anti-CSP serum antibodies from both strains recognized CSP on the surface of the *P*. *yoelii* sporozoite by immunofluorescence microscopy (Fig 1C), indicating that both strains developed antibodies capable of recognizing surface displayed CSP on the sporozoite. However, only BALB/cJ anti-CSP pAbs induced a CSP reaction on sporozoites (Fig 1C, arrows), in which neutralizing antibodies (NAbs) induce the sporozoite to “shed” its coat of CSP [20–22]. We then characterized *in vitro* inhibitory activity in the inhibition of Sporozoite traversal and invasion assay. *Py*CSP-immunized BALB/cJ and C57BL/6J serum antibodies from Weeks 3 and 7 were pre-incubated with *Py* sporozoites and the percentage invasion and traversal to hepatocytes were analyzed. *Py*CSP-immunized BALB/cJ immune serum from both weeks 3 and 7 significantly inhibited sporozoite invasion into hepatocytes compared to controls, whereas *Py*CSP-immunized C57BL/6J immune serum from weeks 3 and 7 did not inhibit invasion above background levels (Fig 1D). *Py*CSP-immunized BALB/cJ pAbs significantly reduced sporozoite traversal at weeks 3 and 7 through hepatocytes compared to controls, whereas *Py*CSP-immunized C57BL/6J immune serum did not inhibit traversal above background levels at any time (Fig 1E). This differential ability to respond to CSP extended to responses against human malaria (*P*. *falciparum*) *Pf*CSP, as pAbs from *Pf*CSP-immunized BALB/cJ and C57BL/6J mice showed similar functional trends. The pAbs from immunized BALB/cJ mice were able to significantly inhibit the invasion of *P*. *falciparum* sporozoites *in vitro*, whereas pAbs from immunized C57BL/6J mice showed little or no inhibition (S2 Fig), indicating that the features defining protection in each strain are generalized and not specific to the species of malaria parasite.

To evaluate the differential functional activity *in vivo*, and to assess potential infection differences due to mouse strain, we performed a pAb swap infection challenge and sterile protection experiment. BALB/cJ or C57BL/6J mice were immunized with *Py*CSP as shown in Fig 1A and then protein A purified pAbs from immunized (Post 3^rd^ immunization) animals or naïve controls were passively infused into strain mis-matched naïve mice five days prior to mosquito bite challenge (15 mosquitoes/mouse). Mice were monitored for two weeks or until blood-stage patency was observed. Thus, C57BL/6J immune and naïve serum was infused into naïve BALB/cJ mice prior to challenge, and vice versa. The pAbs were purified from pooled serum and the anti-CSP content was quantified using a standard curve generated with canonical anti-*Py*CSP mAb, 2F6. Each animal received an equivalent of 90 μg of polyclonal anti-CSP antibody, with naïve animals receiving 90 μg total IgG. C57BL/6J mice that received BALB/cJ-derived anti-CSP pAbs exhibited delays to patency and 40–60% sterile protection, depending on the experiment (Figs 1F and S1B), whereas BALB/cJ mice infused with C57BL/6J-derived anti-CSP pAbs were not protected and all became infected similar to controls (Figs 1F and S1B). These findings demonstrate that the protection observed in vaccinated BALB/cJ mice is mediated by antibodies. Further, it implies that there are specific features of vaccine-elicited antibodies in BALB/cJ mice that are critical to achieving sterile protection, features that are suppressed or do not exist in C57BL/6J mice. This differential model of sterile protection from malaria afforded the opportunity to assess what characteristics of the B cell response are associated with protection from infection.

### *Py*CSP immunized C57BL/6J mice exhibit early deficits in the development of anti-CSP antibodies

**Fig 2:**
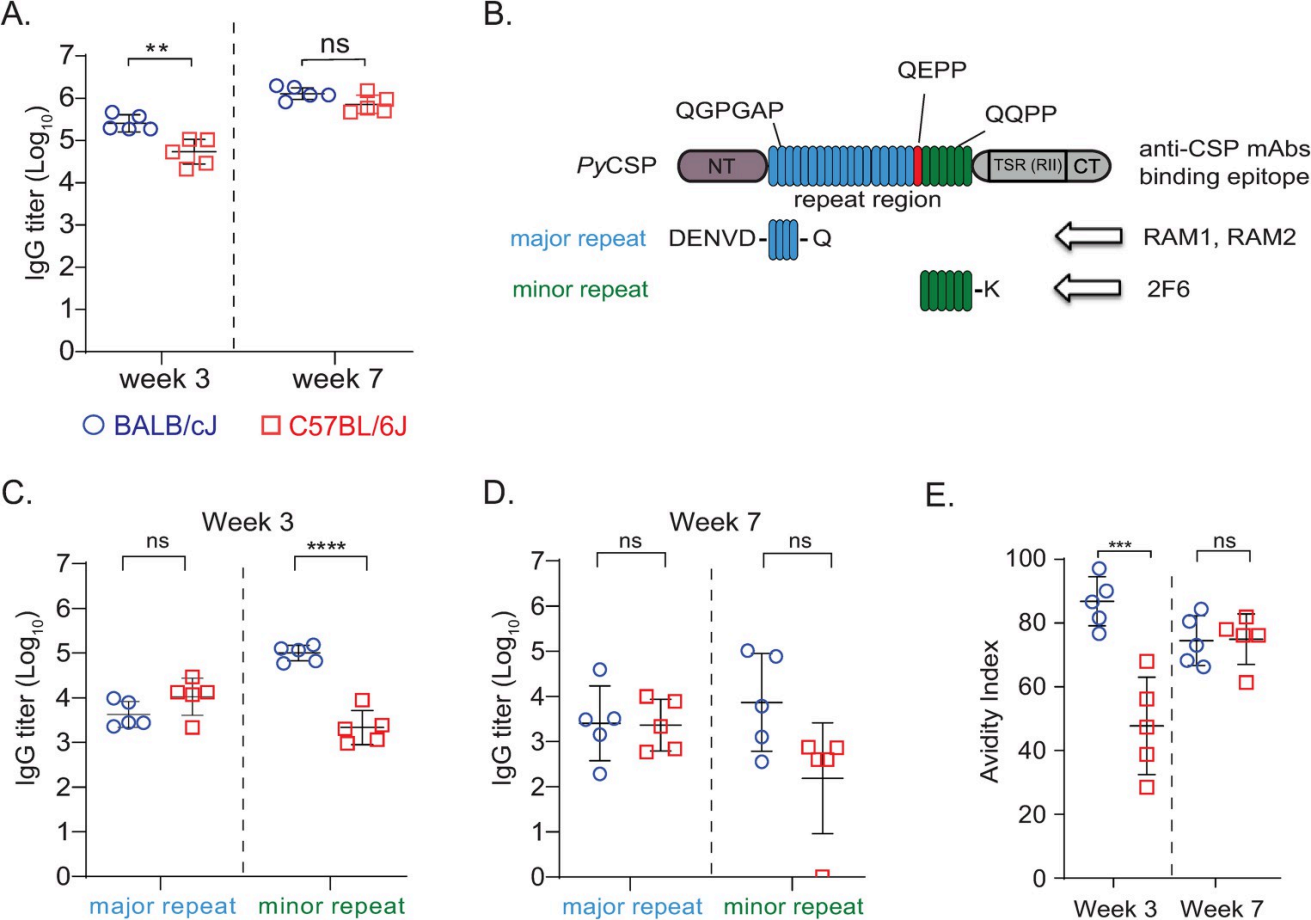
Comparison of antibody responses after immunization of *Py*CSP at week 3 and 7 time points in BALB/cJ and C57BL/6J mice. **A.** 6–8 weeks old female BALB/cJ (n = 5) (blue) and C57BL/6J (n = 5) (red) mice were intramuscularly immunized with 20 μg of *Py*CSP with 20% adjuplex on weeks 0, 2 and 6 and the blood samples were collected on week 3 and week 7. ELISA was performed to analyze the *Py*CSP-specific total IgG titers. **B.** Cartoon representing the domain organization of mature *Py*CSP ectodomain. The N- and C-terminal domains are represented as NT and CT, respectively. The TSR (RII) domain is in CT. The major and minor repeats of the repeat domain are colored blue and green, respectively and their respective amino acid repeat sequences are illustrated. The QEPP amino acid sequence connecting the major and minor repeat regions is indicated in red. The binding epitopes of anti-*Py*CSP mAbs RAM1, RAM2 and 2F6 are indicated with arrows. **C** and **D.** *Py*CSP-specific IgG titers of *Py*CSP-immunized BALB/cJ (blue) and C57BL/6J (red) mice to the major and minor repeats at Week 3 **(2C)** and Week 7 **(2D)** were analyzed**. E.** The avidity of the *Py*CSP-immunization elicited antibodies to the *Py*CSP antigen at weeks 3 and 7 in BALB/cJ (blue) and C57BL/6J (red) mice were analyzed. Avidity index is calculated by (AUC of NH_4_SCN wells)/ (AUC of PBS wells)*100. Data analyzed by Two-way ANOVA and p values were obtained by Tukey’s multiple comparison test. ****p<0.0001; ***p<0.0004 **p<0.*005;* ns- not significant.

To investigate the underlying differences in the protection between *Py*CSP-immunized BALB/cJ and C57BL/6J mice, we analyzed *Py*CSP-specific plasma IgG responses over the course of vaccination. Antibodies are often not measurable after the first immunization but reach high levels with the anamnestic response after the boost immunizations. Thus, we focused on studying samples from after the second and third immunizations, at weeks 3 and 7, respectively. At week 3, α-CSP IgG endpoint titers were nearly one log_10_ higher in immunized BALB/cJ mice than in C57BL/6J mice (Fig 2A). However, by week 7, one week post third immunization and just before mosquito-bite challenge, IgG titers were similar between the strains (Fig 2A). No difference in IgG subclass usage was observed at week 7, and both strains had similar α-CSP titers of IgG1 and IgG2b antibodies (C57BL/6J do not express Ig2a) (S3 Fig). Thus, although we detected an early difference in overall binding titers, at the time of challenge the strains had similar levels and subclass distribution of circulating α-CSP IgG antibodies.

We next measured the avidity index of the serum IgG to full ectodomain *Py*CSP over the course of immunization, which is a surrogate measure of the strength of polyclonal antibody binding to the protein. Binding is measured in the presence and absence of thiocyanate chaotrope, and the relative disruption of binding is used to generate an index value [23]. Interestingly, antibodies from C57BL/6J mice had significantly lower avidity for recombinant *Py*CSP after the second immunization at week 3 (Fig 2E), indicating a potential early deficit in antibody maturation. By week 7 the avidity indices against recombinant *Py*CSP were statistically similar. However, this lack of difference in polyclonal avidity at week 7 is likely due to the coarse measure of the binding characteristics of a diverse mixture of antibodies as a population, where the potential presence of less frequent high affinity clones may not be sufficient to influence the overall avidity index.

To determine whether differential protection could be attributed specifically to epitope specificity, we generated repeat peptides corresponding to the major and minor repeat regions (Fig 2C) and assessed IgG binding over the course of immunization. The repeat regions are known to be the target of neutralizing antibodies, whereas the N- and C-terminal domains have not been implicated as targets for neutralizing antibodies [24–28]. The repeat region in *Py*CSP contains two general motifs that make up the longer, *N*-terminal major repeat region and the shorter, more C-terminal minor repeats (Fig 2B). The minor repeat region is the target of potent α-*Py*CSP neutralizing activity of mAb 2F6 [29–31] but neutralization by mAbs targeting the major repeat region has not been reported. At week 3, the strains had equivalent IgG titers to the major repeat peptide, but BALB/cJ mice had significantly higher titers to the minor repeat peptide (Fig 2D). At week 7, IgG titers to the major repeat remained statistically similar, but, although the difference was not statistically significant, C57BL/6J titers to the minor repeat trended lower (Fig 2D). These findings suggested that the unprotected phenotype in C57BL/6J mice could be due to dampened antibody responses to the minor repeat, which is known to mediate neutralization.

### Both major and minor repeat motifs of *Py*CSP mediate sterile protection from *P*. *yoelii* infection

**Fig 3:**
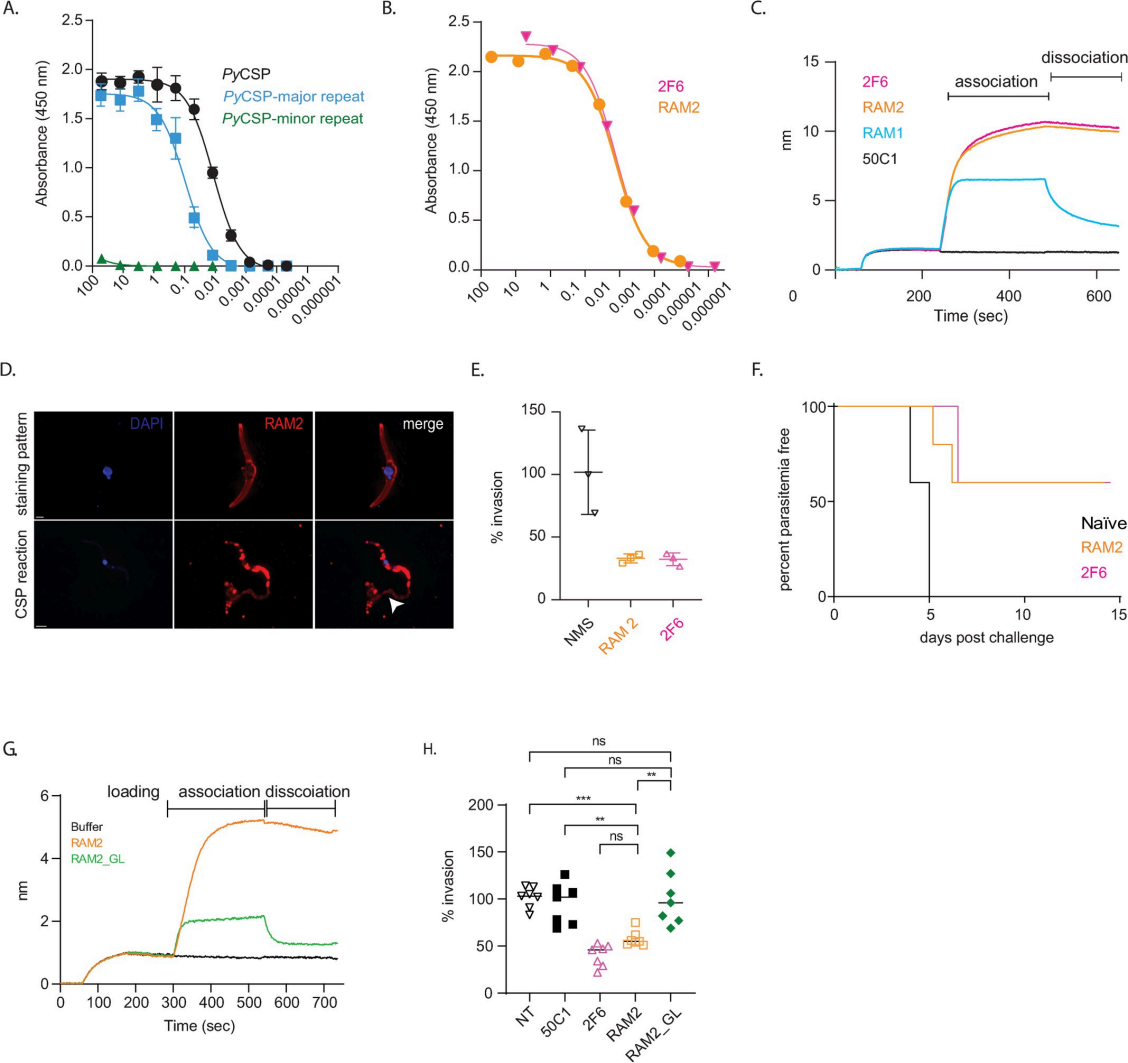
*Py*CSP mAb RAM2 avidly binds to major repeat regions of *Py*CSP and provides sterile liver stage protection from the pathogen. **A.** ELISA showing the binding of RAM2 to the *Py*CSP full length protein (black), the major repeat peptide (blue) but not to the minor repeat peptide (green). **B.** ELISA showing the EC_50_ of RAM2 (orange) and 2F6 (pink) binding to *Py*CSP full length protein The ELISA data in Fig 3A and 3B are derived from three technical replicates. **C.** Biotinylated-*Py*CSP (5 μg) derivatized streptavidin biosensors were incubated in different mAbs (RAM1 (cyan), RAM2 (orange) 2F6 (pink) and 50C1 (black), 5 μg each) and the association and dissociation kinetics were assayed by Octet-BLI **D.** Representative immunofluorescent images of *P*. *yoelii* sporozoites incubated with mAb RAM2 (10 μg) for 10 min. *Py*CSP mAb, 2F6, is used as control for CSP trailing assay and DAPI is used to stain the nucleus of the sporozoites. The arrowhead points to the regions of CSP reaction or shedding (CSPR). **E.** Freshly isolated sporozoites were pre-incubated with (10 μg) of RAM2 (orange), 2F6 (pink), and normal mouse serum (black) antibodies for 10 mins. Hepa 1–6 cells were infected with antibody-treated sporozoites for 90 mins to assess hepatocyte entry. The data here represents the percentage of hepatocytes that were CSP-positive as evaluated by flow cytometry. The data represents mean values ± SE from three independent experiments: n = 3. p values were determined by comparing each treatment to untreated using one-way ANOVA for multiple comparisons tests. **F.** BALB/cJ were injected with 150 μg of RAM2 (orange) or 2F6 (pink) intraperitoneally. Naïve BALB/cJ (black) mice injected with PBS is used as control. After 24 h, mice were challenged with bites from fifteen *P*. *yoelii* infected mosquitoes and patency was assessed from day 4 through day 14. Kaplan-Meier survival plot represents percentage parasitemia free mice over time, including 10 mice from 2 independent experiments. **G.** Biotinylated-*Py*CSP (5 μg) derivatized streptavidin biosensors were incubated in different mAbs (RAM2 (orange), RAM2_GL (green) and 10xkb buffer (black), 5 μg each) and the association and dissociation kinetics were assayed by Octet-BLI. **H.** Freshly isolated sporozoites were pre-incubated with (10 μg) of RAM2 (orange), 2F6 (pink), RAM2_GL (green), 50C1 (black squares) and normal mouse serum (black inverted triangles) antibodies for 10 mins. Hepa 1–6 cells were infected with antibody-treated sporozoites for 90 mins to assess hepatocyte entry. The data here represents the percentage of hepatocytes that were CSP-positive as evaluated by flow cytometry. p values were determined by comparing each treatment to untreated using one-way ANOVA for multiple comparisons tests.

To evaluate this possibility, we investigated whether the major repeat also could be a target for NAbs, or whether neutralization is solely mediated through the minor repeat motif as observed for mAb 2F6. We previously characterized a non-neutralizing antibody (nNAb), RAM1, which binds to the major repeat with relatively low affinity, does not induce the CSP reaction, nor protects *in vitro* or *in vivo* [31]. In this study, we isolated a mAb RAM2, which also binds to an epitope in the major repeat motif like RAM1 (Figs 3A and S4A). IgH and IgK gene usage, as well as CDRH3 characteristics of the mAbs are summarized in S1 Table. RAM2 bound to recombinant CSP with a similar EC_50_ to that of 2F6 by ELISA (Fig 3B), and both mAbs had similar binding kinetics measured by Octet-BLI (Fig 3C). In contrast to RAM1, RAM2 induces the CSP reaction (Fig 3D) and blocks *in vitro* infection to similar levels the NAb 2F6 (Fig 3E). Unlike RAM1, RAM2 also facilitates sterile protection *in vivo* when administered by passive infusion at survival levels that mirror 2F6 (Fig 3F), indicating that potent neutralization can target either the major or minor repeat motifs, and that the differential protection between C57BL/6J and BALB/cJ mice strains cannot be fully explained by differential antibody responses to the major and minor repeat motifs.

Interestingly, although RAM1 and RAM2 both target the major repeat domain, they do so with drastically different binding kinetics. RAM2 Fab binds to recombinant CSP with a K_D_ 7.85±0.585 × 10^−7^ M and with an association and dissociation kinetics values of *k*_on_ (1.25±0.032 × 10^4^ M^-1^s^-1^) and *k*_off_ (9.84±0.083 × 10^−3^ s^-1^), whereas RAM1 Fab exhibits little binding to CSP and we were unable to derive a measurable K_D_ (S4B Fig; [31]). By comparison, the minor repeat NAb 2F6 Fab binds to rec-*Py*CSP with a K_D_ of 5.24 × 10^−7^ M [31]. The difference in binding affinity between RAM1 and RAM2 is likely a major contributor to their differential functional activity, potentially driven by differences in somatic hypermutation (SHM). To test this directly, we reverted RAM2 to its predicted germline precursor and expressed the RAM2-GL antibody. RAM2 possesses 8 amino acid substitutions (5 in IgG and 3 in IgK) from germline resulting from 19-nucleotide changes (14 in IgG and 5 in IgK). The germline reverted RAM2_GL bound poorly to the *Py*CSP antigen with a slower association rate and a faster dissociation rate than RAM2 (and similar to RAM1) indicating that the high affinity of RAM2 is due to its somatic mutations (Fig 3G). Unlike RAM2, RAM2_GL did not inhibit hepatocyte invasion *in vitro* (Fig 3H), clearly demonstrating that the neutralizing activity of RAM2 is dependent on SHM. These findings raise the possibility that the lack of neutralization in C57BL/6J mice may be due to a lack of high affinity CSP-specific B cell clones, potentially driven by lower B cell maturation. Taken together, these findings strongly implicate CSP-specific antibody affinity as a key mediator of protective antibody function against *Plasmodium*.

### Dampened B cell responses elicited by vaccination in C57BL/6J mice

**Fig 4:**
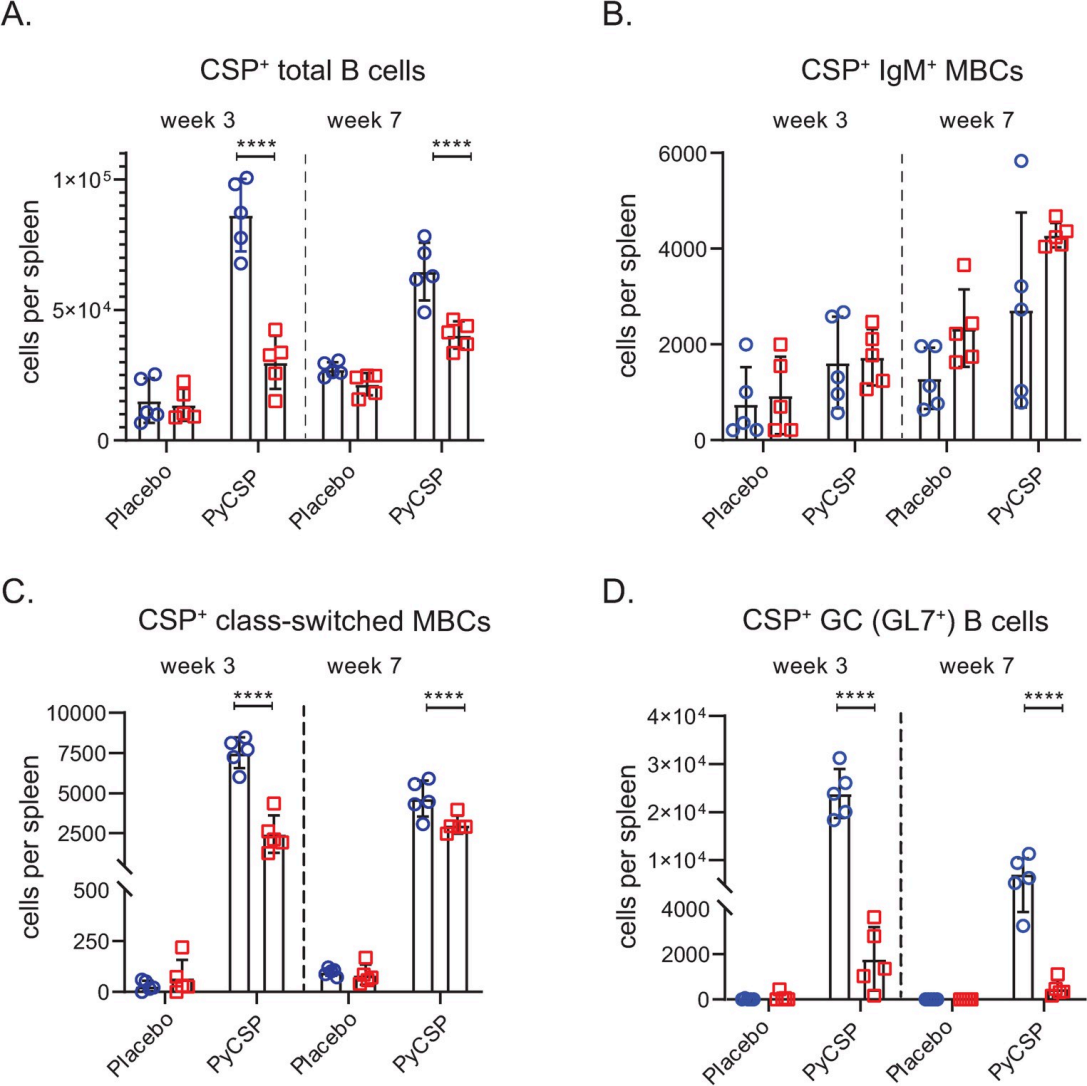
*Py*CSP-specific B-cell responses of BALB/cJ and C57BL/6J mice at weeks 3 and 7. 6–8 weeks old female BALB/cJ (n = 5) and C57BL/6J (n = 5) mice were immunized intramuscularly with 20 μg of *Py*CSP adjuvanted with 20% adjuplex on weeks 0, 2 (Week 3) or weeks 0, 2 and 6 (week 7) and the splenocytes were isolated either on week 3 or week 7 as described in the materials and methods. Cartoon representing the general workflow in B-cell population analysis is illustrated (S5 Fig). B-cells responses of different B-cell populations per spleen were quantified for *Py*CSP-specific B-cells **(A)**, IgM memory B-cells (MBCs) **(B)**, class-switched (sw) Ig+MBCs **(C)**, and Germinal center (GC) B-cells **(D**) in immunized and control BALB/cJ (blue circles) and C57BL/6J (red squares) mice. Data analyzed by Two-way ANOVA and p values were obtained by Tukey’s multiple comparison test. ****p<0.*0001*.

We next evaluated if differential antibody protection between BALB/cJ and C57BL/6J mice was a consequence of nuanced responses to *Py*CSP vaccination in the B cell compartment. To assess the potential immunological origins of the differential protection, we characterized anti-CSP-specific B cell responses in the spleen after vaccination. Splenocytes were harvested at week 3 and 7 or post 1 week after the second and third immunizations, respectively, and stained with markers for flow cytometric analyses, including B220, GL-7, CD38, IgD, IgM, and CD138. Recombinant CSP was tetramerized by conjugation to streptavidin-conjugated APC and APC/Fire750, which were used for column enrichment and dual staining for *Py*CSP-reactive cells. We assessed and quantified the total number of splenic *Py*CSP-specific B-cells (CD3^-^, B220^+^, CSP^+^), IgM memory B-cells (MBCs) (CD3^-^, B220^+^, CD38^+,^ CD138^-^, IgM^+^, IgD^-^, CSP^+^), germinal center (GC) experienced B cells (CD3^-^, B220^+^, GL7^+^, CSP^+^), and class-switched MBCs (CD3^-^, B220^+^, CD38^+,^ CD138^-^, IgM^-^, IgD^-^, CSP^+^) (Figs 4 and S5). Both immunized animals and strain-matched placebo immunized animals were analyzed.

Interestingly, *Py*CSP-immunized BALB/cJ mice exhibited significantly higher numbers of total splenic *Py*CSP-specific B-cells after the 2^nd^ and 3^rd^ immunizations (Fig 4A), indicating a more robust anamnestic response than in vaccinated C57BL/6J mice. This difference was especially apparent in week three, which follows the second immunization, where CSP-specific B cells were nearly 3-fold higher. We did not detect differences in the frequency of CSP^+^ IgM MBCs after either immunization (Fig 4B), although their frequency was relatively low in all samples. However, *Py*CSP-immunized BALB/cJ had more than 3-fold higher numbers of class-switched CSP^+^ MBCs at week three after the second immunization and statistically higher numbers at week 7 (Fig 4C). Strikingly, BALB/cJ mice had approximately 10-fold higher numbers of splenic GL7^+^, GC-experienced CSP-specific B cells than C57BL/6J mice after the second immunization (week 3), and approximately 5-fold more after the third immunization (week 7) (Fig 4D). Thus, except for IgM^+^ MBCs, BALB/cJ mice exhibited comparatively higher *Py*CSP-specific B cell responses, GC activity, and class switch in response to vaccination. As these timepoints measure the anamnestic response, these findings also indicate marked differences in recall responses between the strains. The relative paucity of GC-experienced B cells and class switched MBCs in C57BL/6J mice is intriguing, and likely explains the early differences in IgG titers and binding avidity observed after the second immunization. Overall, the relative deficit of GC activity and class switched MBCs may have contributed to the inability of vaccinated C57BL/6J mice to produce antibodies that can mediate sterile protection from infection.

### *Py*CSP-immunized BALB/cJ mice achieve higher levels of somatic mutation in response to vaccination

**Fig 5:**
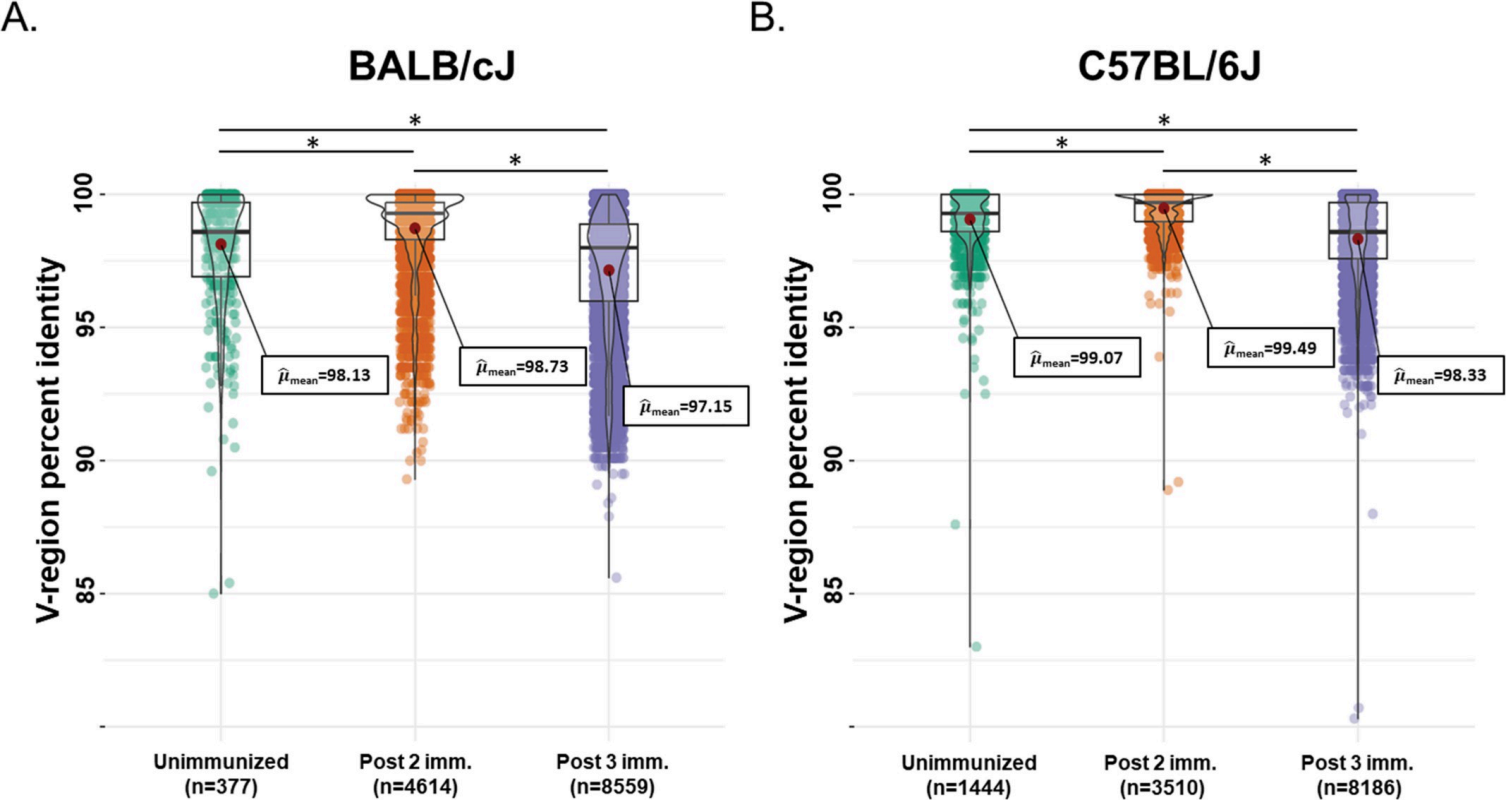
*Py*CSP-immunized BALB/cJ mice generate more highly diverse gamma-chain rearrangements than do C57BL/6J mice. V-regions of IgG heavy chains were amplified from isolated memory B cells of BALB/cJ **(A)** and C57BL/6J **(B)** mice and sequenced. Sequence libraries obtained from unimmunized mice (green) are shown in comparison to those derived from *P*yCSP-specific mouse memory B-cells at Week 3 (orange, post 2^nd^ immunization) and 7 (purple, post 3^rd^ immunization). The data shown are a representative set from three independent experiments. Each data point represents a deduplicated sequence (i.e., a group clustered at 100% sequence identity) and supported by at least 10 raw reads. The visualizations were analyzed and generated using the ggstatsplot (version 0.7.2) R package.

It is possible that the differences in germinal center activity affect overall maturation and somatic mutation within the CSP-specific B cell receptor (BCR) repertoire, which ultimately drives the generation of high affinity antibodies after vaccination. To assess this, we sorted class-switched CSP-reactive MBCs after either two or three immunizations with recombinant *Py*CSP and sequenced the heavy chain BCR repertoire by 5’ Rapid amplification of cDNA ends (5’ RACE) next generation sequencing. Class-switched CSP-specific MBCs (CD3^-^, B220^+^, IgM^-^_,_ IgD^-^, CD38^+^, anti-CSP^+^) were stained and enriched as described above, and then sorted into RLT lysis buffer by fluorescent activated cell sorting (FACS). Class-switched MBCs (CD3^-^, B220^+^, IgM^-^_,_ IgD^-^, CD38^+^) from unimmunized animals were also sorted to serve as a reference for sequence analysis. The sorted, CSP-specific MBCs in mice within each strain were combined, and then γ-chain transcripts were amplified by 5’ RACE RT-PCR and sequenced on an Illumina MiSeq instrument, allowing the retrieval of the entire V, D, J rearrangement. The resulting sequences were processed using our in-house bioinformatics pipeline, which includes V/D/J annotation against a database of germline sequences that included BALB/c- and C57BL/6-derived sequences (22% BALB/c, 46% C57BL/6, 32% other). We then determined the percent identity for each BCR to germline allele segments (to infer the rates of somatic mutation) and third complementarity determining region of BCR heavy chains (CDRH3) characteristics. Higher percent identity to germline indicates less mutation, and conversely, lower identity indicates higher rates of mutation. This analysis was performed for the CSP-specific BCR repertoire at weeks three and seven (i.e., one week following 2^nd^ or 3^rd^ immunization, respectively), as well as the total class-switched MBC γ-chain BCR repertoire from unimmunized animals (Fig 5). The CDRH3 size distributions of the sequenced γ-chain repertoires are shown in S6 Fig.

The average CSP-specific MBC V-region identity after two immunizations in BALB/cJ mice was 98.73%. After three immunizations, identity dropped significantly to an average of 97.15%, perhaps an indicator of higher levels of affinity maturation and somatic mutation stimulated by the three immunizations (Fig 5A). BCR sequences after three immunizations were detected with as low as ~86% identity, and numerous sequences were identified with less than 92.5% identity. Thus, IgG heavy chains with 7.5–14% somatic mutation were selected in response to vaccination, although these were in the minority in the overall repertoire. The identity to germline of CSP-specific MBCs was statistically higher after two immunizations than the average identity of MBCs in unimmunized BALB/cJ mice (98.73% vs. 98.13%), but lower after three immunizations (98.13% vs 97.15%). Thus, on average, compared to the total MBC repertoire in unimmunized animals, CSP-specific MBCs had less mutation after two immunizations, a possible consequence of BCR clonal selection, but significantly more mutation after three immunizations, suggesting ongoing diversification and affinity maturation.

In contrast, the average identity of CSP-specific IgG heavy chains in C57BL/6J mice was 99.49% after two immunizations and 98.33% after three (Fig 5B), with only very rare outliers detected with more than 7.5% mutation. Thus, the CSP-specific IgG heavy chain repertoire in C57BL/6J mice after two immunizations remained close to germline identity, possibly related to the lack of GC experienced CSP-specific B cells detected at this timepoint (Fig 4D). Of note, the non-CSP MBC BCR repertoire in unimmunized mice was more diverse in BALB/cJ mice than in C57BL/6J mice (98.13% vs 99.07), which may be indicative of intrinsic underlying differences between the strains. Although annotation may introduce some artifact due to strain mismatch, the extent of the differences we observe in mutation rates between strains cannot be explained by these differences alone. Taken together, these findings indicate that BALB/cJ mice appear to generate higher levels of somatic mutation in response to vaccination with *Py*CSP. It follows that the higher level of mutation more commonly generated in BALB/cJ mice could be a key factor in their ability to generate high affinity anti-CSP antibodies and achieve sterile protection from infection.

## Discussion

Defining the features of antibodies that can mediate protection from pre-erythrocytic malaria infection would be a key milestone that would guide future vaccine development efforts. As RTS,S rolls out more widely, and newer protein-based vaccines are developed, such as R21, it is essential to understand the desirable characteristics of vaccine-elicited antibody responses that are associated with protection from liver stage infection. Here we studied the characteristics of vaccine-induced responses to recombinant CSP, identifying key differences that implicate B cell maturation as a defining factor in the development of protective antibody responses against sporozoite infection. While these findings are currently correlative and should be confirmed with mechanistic studies, they potentially have direct implications for vaccine development, highlighting the urgent need for vaccine formulations and regimens that drive potent, mature antibody responses.

Previous studies have established that binding affinity to CSP is a key factor in the ability of monoclonal antibodies to neutralize infection [7,13,31–33]. High affinity antibodies are generated through the process of affinity maturation. This occurs in the primary and secondary lymphoid tissues, where antigen-reactive B cells proliferate and migrate into germinal centers. Once in germinal centers, they undergo rounds of somatic hypermutation and clonal selection, ultimately resulting in high affinity B cell clones that secrete antibodies. The effect of affinity on neutralization is exemplified by the mAbs RAM1 and RAM2. RAM2 is protective, whereas RAM1 is not, despite binding to the same repeat motif. Affinity appears to be the major factor driving the difference that determines neutralization; RAM2 binds with an affinity several orders of magnitude higher than RAM1. We confirmed this critical role for somatic mutation by reverting RAM2 to its unmutated germline state (RAM2_GL), which abrogated its high affinity binding and ability to neutralize infection. Our observation of more somatic mutation in the BCR repertoire of BALB/cJ mice also supports the critical role for SHM in protective antibody responses.

Thus, the significant lack of germinal center experienced B cells in C57BL/6J mice is striking, as it indicates that this process is either impaired or happening far less frequently than in BALB/cJ mice [34]. These observations dovetail with our finding that less somatic hypermutation from germline occurred in C57BL/6J mice within the CSP-specific class-switched MBC compartment, indicating that less B cell maturation occurred after vaccination. In fact, the CSP-specific BCR repertoire population average remained close to germline identity, even after the anamnestic response of the second immunization. The natural consequence of less B cell maturation is likely that fewer mature high affinity BCR clones are generated after vaccination, or that overall, the population may be less mutated, as we observed. This is also evident in the lower polyclonal avidity index after the second immunization in C57BL/6J mice, which may be a significant underlying factor in the lack of antibody protection. Each of these observations support the model where less efficient germinal center activity and class switch (and, in turn, insufficient high-affinity CSP antibodies) in C57BL/6J mice leads to a lack of sterilizing protection, whereas robust affinity maturation activity in BALB/cJ mice, resulting in a subset of high-affinity NAbs like RAM2 and 2F6, leads to sterilizing protection.

The underlying immunological origins of reduced germinal center activity in C57BL/6J mice remain unclear and could relate to potential differences in MHC alleles or subpar T helper responses. However, vaccine protection from *P*. *berghei* rodent malaria infection has been reported in C57BL/6J mice, especially with adjuvants that strongly drive B cell development, such as Matrix M [35]. Further, in this study passive transfer of CSP-specific antibodies elicited in BALB/cJ mice achieved sterile protection in C57BL/6J mice. This indicates that it is possible to elicit or passively transfer antibodies in C57BL/6J mice that mediate sterile protection. As such, it is likely that our vaccine regimen was not sufficient to elicit antibodies with the desired characteristics in C57BL/6J, despite eliciting very high titers of anti-*Py*CSP antibodies. Therefore, it is not that C57BL/6J mice were generally hypo-responsive to vaccination or overly sensitive to infection, but rather a deficit in maturing the B response toward ideal neutralization characteristics likely underlies the total lack of protection [34]. Thus, although we do not know the underpinnings of the differential antibody responses in C57BL/6J mice, comparative use of this model allowed the identification of fundamental immunological processes that could be key parameters for achieving durable sterilizing immunity. However, our findings in the polyclonal response remain correlative with protection, and the role of B cell maturation should be confirmed directly, potentially through the immunization and challenge of mouse strains impaired for GC activity. Further, while the differential protective activity also extended to *P*. *falciparum in vitro*, and CHMI studies of human mAbs corroborate our findings, it would be prudent to evaluate this phenomenon against human-infective malarias *in vivo*.

Eliciting durable, potent antibody responses by vaccination is a considerable challenge for the field. RTS,S, which has just been approved for wide usage by the WHO, contains the potent adjuvant AS01, elicits high titers of CSP antibodies, but titers and partial protection wanes quickly after vaccination [8]. R21, which is similar to RTS,S, is currently in testing with the potent adjuvant Matrix M, which is hoped to be superior in eliciting high titers of antibody responses [8]. However, our findings imply that high titers of antibodies alone are not sufficient to achieve potent, durable protection from infection. Rather, it is the quality of the antibodies in the response that will determine whether protection can be achieved. A careful examination of B cell responses, including the extent of their hypermutation and germinal center experience, may be critical for extracting meaningful correlates of protection and, ultimately, designing the most potent vaccine regimen against malaria. Novel approaches that emphasize the elicitation of high affinity antibodies to neutralizing epitopes will likely be a more fruitful path forward for vaccine development. Such a vaccine would dramatically impact our ability to curtail malaria infection worldwide and could make a substantial impact in the push towards eradication.

## Materials and methods

### Ethics statement

All the experiments involving animals were performed in adherence to protocols of Center for Infectious Disease Research Institutional Animal Care and Use Committee (IACUC) and recommendations of the NIH office of Laboratory Animal Welfare standards. All the mice were housed under specific pathogen free conditions at the Center for Global Infectious Diseases Research, Seattle Children’s Research Institute.

### Cloning and production of *Py*CSP and truncation constructs

*P*. *yoelii* CSP ectodomain, a 403-amino acid (aa) protein, consisting of an *N*- and *C*-terminal domains connected by a central repeat domain (Fig 2B). The two predicted *N*-glycosylation sites (S27A, T348A), one each in the N- and C-terminal domains of the native antigen were mutated to prevent the addition of *N*-linked glycans in the mammalian expression system and the non-glycosylated protein, *Py*CSP (38.3 kDa), is used in this study. The cartoon representing the *Py*CSP, and different major and minor repeat peptides used in this work are illustrated in Fig 2B and the construction and expression of the protein is published elsewhere [31]. The *Py*CSP antigen was codon optimized for human bias and C-terminally 8X His and Avi-tagged, connected via a GS-linker to the antigen, to facilitate purification and biotinylation. A tPA signal was added at the *N*-terminus to facilitate protein secretion and the construct was cloned into pcDNA3.4 vector (Thermo Fisher, Waltham, MA, USA) which drives transcription via a CMV promoter. After sequence confirmation the plasmid DNA (500 ng/ml of cells) encoding the antigens were introduced into HEK293 suspension cultures (1 million/ml) by high-density transfection using 2 mg/ml polyethyleneimine (PEI) (1,5 Pei:plasmid DNA). Cells were grown in FreeStyle 293 serum-free media (Thermo Fisher) for five days at 37°C 8% CO_2_. Cells were spun down by centrifugation (4000 rpm for 20 min at 4°C) and the supernatant was collected. Sodium azide was added to a final concentration of 0.02% and NaCl was added to a final concentration of 500 mM. The antigen was purified by a two-step chromatography protocol. The protein was captured using Ni-NTA (Qiagen, Germantown, MD, USA), washed in EQ buffer (25 mM Tris pH 8.0, 300 mM NaCl, 0.02% NaN3), and eluted in EL buffer (25 mM Tris pH 8.0, 300 mM NaCl, 200 mM Imidazole, 0.02% NaN3). The elution fractions were collected, pooled, and concentrated and further purified on a standardized Superdex 200 16/600 (GE Healthcare, Chicago, IL, USA) column running in HBS-E (10 mM HEPES, pH 7, 150 mM NaCl, 2 mM EDTA). Peak fractions were pooled, concentrated, and stored at 4°C until use.

### Animal immunization

All animal studies were conducted under protocols reviewed and approved by the Institutional Animal Care and Use Committee (IACUC) at Seattle Children’s Research Institute. For regular immunization of 6–8 weeks old female BALB/cJ and C57BL/6J mice, 20 μg of *Py*CSP formulated in 20% adjuplex is administered intramuscularly at weeks 0, 2 and 6. Blood samples were collected at one week post 2^nd^ and 3^rd^ immunizations i.e., week 3 and week 7, by submandibular or chin bleeds. Placebo-injected mice were used as experimental control. For studying the B-cell responses and NGS of the antigen-specific memory B-cells, mice were harvested at week 3 or 7 followed by splenocyte isolation. For pathogen challenge experiments, all the mice were exposed to *P*. *yoelii* infected mosquito bite challenge (10–15 mosquitoes/mouse) in week 7 and followed for 2–3 weeks for blood-stage patency. For serum antibodies swap passive transfer experiment, all the naïve BALB/cJ mice were injected i.p. with 90 μg *Py*CSP-antibodies purified from *Py*CSP-immunized C57BL/6J mice and vice versa followed by mosquito bite challenge (10–15 mosquitoes/mouse) 5 days post passive transfer of antibodies. As a control, purified naïve BALB/cJ serum antibodies (90 μg) were i.p. transferred to C57BL/6J mice and vice versa. All the mice were monitored for 2–3 weeks for blood stage patency.

### ELISA

Plasma antibody binding to *Py*CSP and to major or minor repeat peptides were determined using a Streptavidin-capture ELISA. Mouse plasma samples were heat-inactivated for 30 min at 56°C prior to all assays. All ELISA incubations were done for 1 h at 37°C and plates were washed between each ELISA step with PBS, 0.2% Tween-20. To determine antibody binding to the ligand, Immulon 2HB 96-well plates (Thermo Scientific, 3455) were coated with 50 ng/well of Streptavidin (NEB, N7021S) in 0.1 M NaHCO_3_, pH 9.5, followed by blocking with 3% BSA in PBS. Later the plates were coated with 200 ng/well with biotinylated ligand antigen or peptides followed by a blocking step with 10% non-fat milk, 0.3% Tween-20 in PBS. Later mouse plasma was serially diluted in duplicate over a range of 1:200 to 1:55,987,200 in PBS with 0.2% BSA. *Py*CSP mAb, 2F6, was serially diluted on each plate to ensure intra-assay consistency. Bound antibodies were detected using goat anti-mouse IgG Fc-HRP (Southern Biotech) or goat anti-mouse IgG1 Fc-HRP (Southern Biotech) or goat anti-mouse IgG2a Fc-HRP (Southern Biotech) or goat anti-mouse IgG2b Fc-HRP (Southern Biotech) at 1:2000 dilution in PBS with 0.2% BSA. Plates were developed with 50 μl/well of TMB Peroxidase Substrate (SeraCare Life Sciences Inc, 5120–0083) and stopped after 3 min with 50 μl/well of 1 N H_2_SO_4_. Absorbance at 450 nm was read using a BioTek ELx800 microplate reader. Binding curves were generated by nonlinear regression (log[agonist] vs response [three parameters]) in GraphPad Prism V8 (San Diego, CA). All the ELISA results were determined from three technical replicates, unless otherwise mentioned. Endpoint titers were defined as the reciprocal of plasma dilution at OD 0.1.

### Chaotrope dissociation ELISA

Antibody avidity of post-2^nd^ and 3^rd^ (Week 3 and week 7) immunization plasma samples to *Py*CSP were determined using direct immobilization ELISA. Immulon 2HB 96-well plates (Thermo Scientific, 3455) were coated with 50 ng/well of *Py*CSP overnight at room temperature, then blocked the following day with PBS-containing 10% non-fat milk, 0.3% Tween-20 for 1 h at 37°C. Plates were washed between each step with PBS-containing 0.2% Tween-20. Mouse plasma samples were heat-inactivated for 30 min at 56°C prior to all assays. Plasma was diluted in PBS, 10% non-fat milk, 0.03% Tween-20 over a range of 1:50 to 1:9,331,200 and plated in quadruplicate side-by-side on the same plate. After a 1 h incubation with antibodies, plates were washed, and half of the sample wells were treated with 2 M NH_4_SCN in PBS, while the other half was treated with PBS alone for 30 min at room temperature. Plates were then washed and goat anti-mouse IgG Fc-HRP (Southern Biotech, 1013–05) was added to the plate at 1:2000 in PBS, 10% non-fat milk, 0.03% Tween-20. Plates were developed as described above. The avidity index was calculated as the ratio of AUC of samples treated with 2 M NH_4_SCN over AUC of samples treated with PBS: (AUC_NH4SCN_ / AUC_PBS_)×100.

### Monoclonal antibodies isolation and production

The method of generation of *Py*CSP monoclonal antibodies was described earlier [31,36]. Briefly, six weeks old female BALB/cJ mice were immunized with 20 μg of *Py*CSP adjuvanted with 20% v/v of Adjuplex at 0, 2 and 6 weeks followed by spleens harvest and splenocyte isolation at Week 7. Using EasySep mouse B cell enrichment kit and manufacturer’s protocol (StemCell Technologies Inc., Tukwila, WA, USA), B cells were isolated by negative selection and resuspended in FACS buffer (PBS with 2% Fetal calf serum) followed by staining with anti-mouse CD16/CD32 (mouse Fc block; BD Biosciences) and a decoy tetramer (BV510) for 10 mins at room temperature. Later the cells were stained with CD38-APC, IgM-FITC, IgD-AF700, B220-PacBlue (BioLegend, San Diego, CA, USA) and *Py*CSP tetramer (BV786) for 30 mins at 4°C followed by a wash with FACS buffer. Finally, the cells were resuspended in FACS buffer and filtered by using a 30-micron filter followed by single-cell sorting using a BD FACS Aria II with a 100-μm nozzle running at 20 psi. *Py*CSP-specific class switched memory B cells (B220+ CD38+ IgM- IgD- antigen+ decoy- cells) were isolated and sorted as single cell per well into 96-well PCR plates. For the cDNA generation and the consequent IgG and IgK variable region amplification a previously described [37] protocol was followed and the PCR amplified final heavy and light chain sequences were cloned by Gibson assembly into pcDNA3.4 expression vectors containing the murine IgG1 and IgK constant regions, respectively. The IgG and IgK plasmid DNA sequences were verified for the CDR3 sequences by Sanger sequencing and these plasmid pairs were diluted in PBS followed by addition of polyethyleneimine (Polysciences, Warrington, PA). After 15 mins of incubation at room temperature, the mixture was added to HEK293-F cells at a density of 1 million cells per milliliter cultured for 5 days in mammalian FreeStyle at 37°C, 5% CO_2_ on a shaker platform for mAb expression. The cells were separated from the supernatant by centrifugation at 4000 rpm for 20 mins at room temperature and the cell-free supernatant was passed over a HiTRAP MabSelect Sure column (GE Lifesciences #11003493) followed by wash and elution steps performed as suggested by the manufacturer. The isolated mAbs were buffer exchanged into HBS-E (10 mM HEPES, pH 7, 2 mM EDTA, 150 mM NaCl) and the homogeneity and the size of the antibodies were analyzed by using an analytical Superdex 200 10/300 column (GE Healthcare, Chicago, IL, USA).

### Tetramer production

The following tetramers were made for antigen enrichment of splenocytes at an 8:1 ratio of protein to SA-fluorophore, based on protein biotinylation efficiency. Bio-*Py*CSP protein (38.3 kDa) at 16 μM was combined with SA-APC antibody (Biolegend) at 2 μM. Bio-*Py*S23 protein (44.8 kDa) at 16 μM was combined with SA-APC/Fire750 antibody (Biolegend) at 2 μM and was used as a decoy. To achieve optimal binding, an antibody was added to protein in two additions with 20 min incubations at room temperature following each addition.

### Splenocyte isolation and Antigen-specific cell enrichment

Splenocytes were isolated from fresh spleens by passing the tissue through a 70 μm strainer and rinsing with splenocyte buffer (1X phosphate buffered saline (PBS) supplemented with 2% fetal bovine serum (FBS), 100 μg/mL DNase I (Sigma) and 5 mM MgCl_2_. Collected cells were spun down at 350 x g for 10 min and resuspended in the FACS buffer (1X PBS supplemented with 2% FBS). Fc block (BD Biosciences) was added to each sample at a 1:100 dilution with decoy tetramer (Bio-*Py*S23, SA-APC/Fire750) and samples were incubated for 10 min at RT. Then the *Py*CSP tetramer (Bio-*Py*CSP, SA-APC) was added, and samples were incubated for 30 min at 4°C. After the incubation, cells were washed with FACS buffer and spun at 300x g for 10 min at 4°C. Anti-APC microbeads (Miltenyi Biotec) in FACS buffer were added and samples were incubated for 15 min at 4°C. After washing, magnetic separation of cells was performed with LS columns (Miltenyi Biotec) on a quadroMACS separator (Miltenyi Biotec). Cell suspensions were applied to a pre-separation 30 μM filter on separate LS columns. Untetramerized cells without magnetic beads that passed through the column were collected as flow through. Then the columns were removed from the quadroMACS magnet and magnetically labeled cells were flushed out with the FACS buffer and collected. Both the labeled and flow through fractions were spun at 300xg for 10 min and resuspended in the FACS buffer. The gating strategies are shown in S5B Fig.

### Antibody staining and flow cytometry

The following antibody mixture was made and used to stain the flow through and labeled fractions: B220-BV510 (Biolegend) at 1:20, CD38-FITC (Biolegend) at 1:100, IgM-BV786 (BD Biosciences) at 1:20, IgD-PercpCy5.5 (Biolegend) at 1:80, CD3-APC/Fire (Biolegend) at 1:40, GL7-e450 (ThermoFisher) at 1:40, and CD138-BV605 (BD Biosciences) at 1:80. Samples were incubated on ice for 30 min, washed with FACS buffer and resuspended in cold 1% paraformaldehyde (PFA) in PBS and stored overnight at 4°C until collection of events on the cytometer the following day. For compensation controls, cryopreserved splenocytes were removed from liquid nitrogen storage, quickly thawed at 37°C in water bath for 1 min, washed in RPMI media with DNase (RPMI 1640, Corning +10% FBS + 1:100 Pen/strep + 2 mM L-glutamine +100 μg/mL DNase I + 5 mM MgCl_2_), passed through a 70 μM cell strainer, washed again and finally resuspended in FACS buffer. Single stains controls were made with the following antibodies: B220-BV510 (Biolegend) at 1:20, CD38-FITC (Biolegend) at 1:100, IgM-BV786 (BD Biosciences) at 1:20, IgD-PercpCy5.5 (Biolegend) at 1:80, CD3-APC/Fire (Biolegend) at 1:40, B220-APC (Biolegend) at 1:50, B220-e450 (ThermoFisher) at 1:33, and B220-BV605 (Biolegend) at 1:20. Samples were incubated for 25 min at 4°C then washed with the FACS buffer and resuspended in cold 1% PFA in PBS and stored at 4°C overnight. In the flowcytometry experiments to evaluate the number of specific B-cell populations. 1% PFA was added to a small portion of unstained, labeled and flow-through cells and reserved for cell count analysis. These samples were incubated overnight at 4°C followed by the addition of Accucheck beads (ThermoFisher) the following day and the events were collected on the cytometer. For quantification of specific B-cells an LSR II flow cytometer (BD Biosciences) was used, 1.5–2.5 million events were collected for each sample at a threshold of 15,000. A total of 50,000 events were collected for cell count analysis. All the data were analyzed using FlowJo software (FlowJo LLC).

### Biolayer interferometry (BLI)

Biolayer interferometry (BLI) is used to test the antigen, antibody interactions using an Octet QK^e^ instrument (ForteBio Inc., Menlo Park, CA, USA). Streptavidin (SA) biosensors (ForteBio Inc.,) were used to immobilize the biotinylated antigen or peptide, followed by incubation in 10X Kinetics Buffer (PBS+0.1% BSA, 0.02% Tween-20 and 0.05% sodium azide). The antigen-derivatized probes were then dipped in indicated concentrations of antibodies in 10X Kinetics Buffer, respectively, followed by an incubation step in 10X Kinetics buffer to test the dissociation of the interactions. The resulting sensograms from the association and dissociation phases were normalized to the buffer values and analyzed by a global fit simple 1:1 binding model using the ForteBio data analysis software (version 7.0.1.5). The K_D_ was determined from the estimated on- and off-rates of the samples.

### Fab generation

The RAM2 Fabs were generated by papain digestion and purified using Immobilized papain agarose resin (Thermofisher Scientific) following manufacturer’s protocol. Briefly 1 mg of RAM2 mAb in 400 μl of sample buffer (20 mM Sodium phosphate pH 7.0, 10 mM EDTA) is mixed with 400 μl immobilized papain resin in digestion buffer (20 mM Sodium phosphate pH 7.0, 10 mM EDTA, 20 mM Cysteine.HCl) and incubated overnight on a rotatory shaker at 37°C. Later 1.5 ml of Tris-HCl, pH 7.5 is added to the mixture followed by centrifugation at 2000 x g for one minute at room temperature. The supernatant containing Fabs is separated from the immobilized agarose resin and is passed through a 0.22 μM spin column. Finally, the Fab fragments were isolated by negative selection on a HiTrap MabSelect SuRe cartridge with 10 column volumes of PBS, pH 7.4 and concentrated on spin column to desired concentration.

### Generation of infected *Anopheles stephensi* mosquitoes

*P*. *yoelii* wild type strain 17XNL (BEI resources) was maintained in Swiss Webster (Envigo, SW) mice. Swiss Webster mice were injected intraperitoneal (i.p.) with 250 μL of infected blood at 3–5% parasitemia. Gametocyte exflagellation rate was checked 4 days post-injection. Mice were then anesthetized with 150 μL Ketamine Xylazine solution (12.5 mg/mL ketamine, 1.25 mg/mL xylazine in PBS) and naïve female *Anopheles stephensi* mosquitoes (3–7 days old) were allowed to feed on them. Mosquitos were maintained at 23°C, 80% humidity with a 12L-12D light cycle. On day ten, the midguts from ten mosquitos were dissected and the number of oocysts was counted as a measure of infection. On day 15 post-infection, the salivary glands of the mosquitoes were dissected to extract sporozoites.

### Mosquito bite challenge

Mice were anesthetized by IP injection of 150 μL Ketamine Xylazine solution (12.5 mg/mL ketamine, 1.25 mg/mL xylazine in PBS). Once anesthetized pairs of mice were each placed on a carton of 15 infectious mosquitoes and mosquitoes were allowed to bite through the mesh top for 10 min. Every 30 sec mice were rotated among mosquito cartons to ensure equal exposure of mice and to maximize mosquito probing thereby sporozoite transfer. Mice received subcutaneous PBS injections and recovered from anesthesia under a heat lamp. Mice were checked for the presence of blood-stage parasites (patency) beginning four days post-challenge.

### Assessment of blood stage patency

Patency was checked daily by blood smear 4 days post-infection by tail snip. Slides were fixed in methanol, dried, and then stained with giemsa (1:5 in H_2_O) for 10 min and viewed at 100X oil immersion on a compound microscope and twenty fields of view examined for each smear. Mice were considered patent if 2 or more ring stage parasites were observed.

### Sporozoite quantification

To quantify the number of sporozoites per mosquito, 10–12 mosquitoes’ salivary glands were hand dissected. These glands were then ground with a pestle and spun at 800 rpm to pellet debris. Sporozoites were counted on a hemocytometer.

### Invasion and traversal assay

Freshly isolated sporozoites were exposed to 2F6, RAM1, RAM2 or serum antibodies from *Py*CSP immunized BALB/cJ and C57BL/6J mice for 10 mins at indicated concentrations. For invasion experiments, 5×10^5^ Hepa 1–6 cells were seeded in each well of a 24-well plate (Corning) and infected with antibody exposed *P*. *yoelii* sporozoites at a multiplicity of infection (MOI) = 0.25 for 90 mins. For traversal experiments 5×10^5^ HFF-1 cells were co-incubated with high molecular mass Dextran-FITC (70 kDa) (Sigma). After 90 mins of infection, cells were harvested with accutase (Life Technologies) and fixed with Cytoperm/Cytofix (BD Biosciences). Cells were blocked with Perm/Wash (BD Biosciences) + 2% BSA for 1 h at room temperature then stained overnight at 4°C with antibodies to CSP- Alexafluor-488 conjugate. The cells were then washed and resuspended in PBS-containing 5 mM EDTA. Infection rate was measured by flow cytometry [38] on an LSRII (Becton-Dickinson) and analyzed by FlowJo (Tree Star).

### Immunofluorescence

Freshly isolated sporozoites were exposed to 2F6, RAM2, RAM1 or serum antibodies from *Py*CSP immunized BALB/cJ and C57BL/6J mice for 10 mins at indicated concentrations. Sporozoites were spun at 17000 x *g* for 5 mins and washed with 1x PBS-EDTA and fixed with 3.7% PFA for 20 mins. Sporozoites were transferred to 8-well chambered slides and the images were acquired with a 100×1.4 NA objective (Olympus) on a DeltaVision Elite High-Resolution Microscope (GE Healthcare Life Sciences). The sides of each pixel represent 64.5×64.5 nm and z-stacks were acquired at 300 nm intervals. Approximately 5–15 slices were acquired per image stack. For deconvolution, the 3D data sets were processed to remove noise and reassign blur by an iterative Classic Maximum Likelihood Estimation widefield algorithm provided by Huygens Professional Software (Scientific Volume Imaging, The Netherlands).

### Next-generation sequencing (NGS)

RNA from CSP-specific MBCs was isolated, gamma-chain transcripts were amplified by 5’ Rapid amplification of cDNA ends (5’RACE) RT-PCR, and the resulting libraries were sequenced on an Illumina MiSeq instrument to yield complete coverage of the variable segment [39]. Briefly, cell lysates from 3–100 × 10^3^ cells were homogenized using QIAshredder columns (Qiagen, #79654). RNA was extracted and purified using AllPrep DNA/RNA Mini kit (Qiagen, #80204), and concentrated using RNAClean XP beads (Beckman Coulter, #A63987). The concentration and quality of the RNA was determined using an Agilent 2100 Bioanalyzer with the Agilent RNA 6000 Pico Kit (Agilent Technologies, #5067–1513). The oligonucleotides used in library construction are shown in S2 Table. Up to 1 μg of RNA was mixed with the gamma-chain reverse primer (vv-534), incubated at 72°C for 3 min to denature the RNA and then cooled to 42°C to allow the primer to anneal. cDNA was generated by mixing 5x First-Strand Buffer, DTT (20 mM), dNTP mix (ThermoFisher, #18427–013, 10 mM), a unique molecular identifier (UMI)-tagged template-switch oligo (TSO) (vv-877), Recombinant RNase Inhibitor (Takara Bio/Clontech, #2313A, 40 U/μL), and SMARTScribe RT (Takara Bio/Clontech, #639536, 100 U/μL) with the denatured RNA/oligo complexes and incubating for 90 min at 42°C then heating to 70°C for 10 min. Uracil-DNA glycosylase (NEB, #M0280S, 5 U/μL) was added, and the reaction was incubated for 1 h at 37°C to degrade the TSO. RNAClean XP beads were used to purify and concentrate the cDNA. Subsequent amplification was performed with primers vv-869, vv-870 and vv-872, using Q5 2x Master Mix (NEB, #M0492S). The reaction was incubated for 30 s at 98°C, then cycled 18 times at 98°C for 10 s, 63°C for 30 s, and 72°C for 30 s, and finally incubated at 72°C for 5 min. Product DNA was isolated using SPRI beads (Beckman Coulter, #B23318), according to the manufacturer’s protocol. Further target DNA enrichment was done via nested PCR using primers vv-873, vv-874 and vv-876 under the same reaction conditions, but with a modified annealing temperature of 60°C for 8–15 amplification cycles. Following a SPRI-bead product cleanup, the libraries were modified by adaptor ligation using the NEBNext Ultra II DNA Library Prep Kit (NEB, #E7645) and indexed using the NEBNext Multiplex Oligos for Illumina (NEB, #E7335). Libraries were quantified using a KAPA Library Quantification Kit (Roche, KK4873) on a QuantStudio3 Real Time PCR System (ThermoFisher, #A28567), pooled to a concentration of 4 nM, denatured in 0.2 N NaOH and diluted to 20 pM. To increase read diversity, PhiX Control V3 (Illumina, #FC-110-3001) was denatured, diluted to 20 pM and added to the library pool as a 1% spike-in prior to loading onto the MiSeq Reagent Kit v3 (Illumina, #MS-102-3003) cassette.

Raw Illumina MiSeq reads were processed using an approach similar to the one previously described [39] with several modifications to utilize UMI-based error correction [40]. The pipeline code is available (https://github.com/vladimirvig/ngs-ig). Briefly, following the amplicon reconstruction and oligonucleotide trimming, UMI sequences were identified and used to collect sequences into molecular identifier groups (MIGs), representing PCR-amplified mRNA molecules. Consensus sequence for each MIG was then determined using the approach adapted from the MIGEC pipeline [41], in which the MIG is first represented by a position frequency matrix, followed by base calling and calculation of a cumulative quality score for each position. Resulting sequence sets were annotated using IgBLAST (version 1.11.0) [42] against a custom database of mouse germline sequences (including BALB/cJ- and C57BL/6J-derived sequences) obtained from the IMGT/GENE-DB collection [43] (www.imgt.org) to determine segment boundaries (e.g., to define CDR3 regions), identify closest germline matches and derive sequence-identity-to-germline values. In order to eliminate multiple identical transcripts likely originating from the same B cell, sequence set deduplication was carried out using VSEARCH [44] (Version 2.9.1) at 100% sequence identity. In the finalized deduplicated data sets, only sequences that were supported by ≥10 raw reads were used in further analysis. The visualizations were generated using the ggstatsplot (version 0.7.2) package in R (version 4.0.2).

## Supporting information

S1 Fig **A.** Graphics showing the statistical significance of *Py*CSP-immunized BALB/cJ (blue), C57BL/6J (red) and their respective placebo controls in green and yellow. **B.** Statistics showing the significance of the delay in blood stage patency in swapped *Py*CSP-pAbs (90 μg)-infused BALB/cJ (blue), C57BL/6J (red) and their respective placebo controls in green and yellow. The days to patency are measured and the number of mice that are sterile protected in the *Py*CSP-immunized BALB/cJ mice **(A)** and *Py*CSP-immunized BALB/cJ mice pAbs infused naïve C57BL/6J mice **(B)** were indicated. Data analyzed by Two-way ANOVA and p values were obtained by Tukey’s multiple comparison test.
(TIF)

S2 Fig Differential *in vitro* invasion inhibition of pAbs from *Pf*CSP-immunized BALB/cJ and C57BL/6J mice. Purified pAbs (1:10) from *Pf*CSP-immunized BALB/cJ (blue) and C57BL/6J (red) mice were assayed for *in vitro* functional activity as described in the Materials and Methods section. A canonical *Pf*CSP mAb-2A10 (grey) and untreated (NT, Black) cells were used as experimental controls.
(TIF)

S3 Fig IgG subclass differences between the *Py*CSP-immunized BALB/cJ and C57BL/6J mice. The plasma IgG1, IgG2a (only for BALB/cJ), IgG2b, IgG2c (only for C57BL/6J) and IgG3 antibody responses of *Py*CSP-immunized BALB/cJ (blue circles) and C57BL/6J (red squares) mice at week 7 (pre-challenge) were measured by ELISA using biotinylated *Py*CSP as ligand. Data analyzed by Two-way ANOVA and p values were obtained by Sidak’s multiple comparisons test. ****p<S0.*0001*.
(TIF)

S4 Fig RAM2 association and dissociation kinetics. **A.** Biotinylated-major repeat peptide (5 μg) loaded streptavidin biosensors were dipped in 5 μg each of mAbs (RAM1-cyan, RAM2-orange, 2F6-pink and 50C1-black) and the association and dissociation kinetics were assayed by Octet-BLI. **B.** Streptavidin biosensors loaded with *C*-terminally biotinylated-*Py*CSP were incubated in different concentrations of RAM2 Fab ranging from 7500 nM to 625 nM and the association and dissociation kinetics were analyzed. The resulting association and dissociation sensograms were analyzed by a global fit 1:1 binding model using the ForteBio data analysis software (version 7.0.1.5) generating K_D_ as estimated from the on- and off-rates.
(TIF)

S5 Fig **A.** Summary of the workflow showing the different steps involved in the quantification of CSP-specific B-cell responses as described in the Materials and Methods section. **B.** Overview of the gating strategy used in Fig 4.
(TIF)

S6 Fig The CDRH3 amino acids (aa) lengths and density of (A) *Py*CSP-specific MBCs γ chain sequences from naïve animals (lavender), and after two (peach) or three (green) immunizations in BALB/cJ mice, and (B) from C57BL/6J mice. The data shown are a representative set from three independent experiments.
(TIF)

S1 Table Gene usage and CDR3 characteristics of anti-CSP mAbs. (DOCX)

S2 Table Oligonucleotides used in the NGS experiment. (DOCX)
