# Supplementary figures and images for: Germinal center activity and B cell maturation are associated with protective antibody responses against Plasmodium pre-erythrocytic infection
Source: PLoS Pathog. 2022 Jul 6;18(7):e1010671. doi: 10.1371/journal.ppat.1010671 (PMC9292112; doi:10.1371/journal.ppat.1010671)

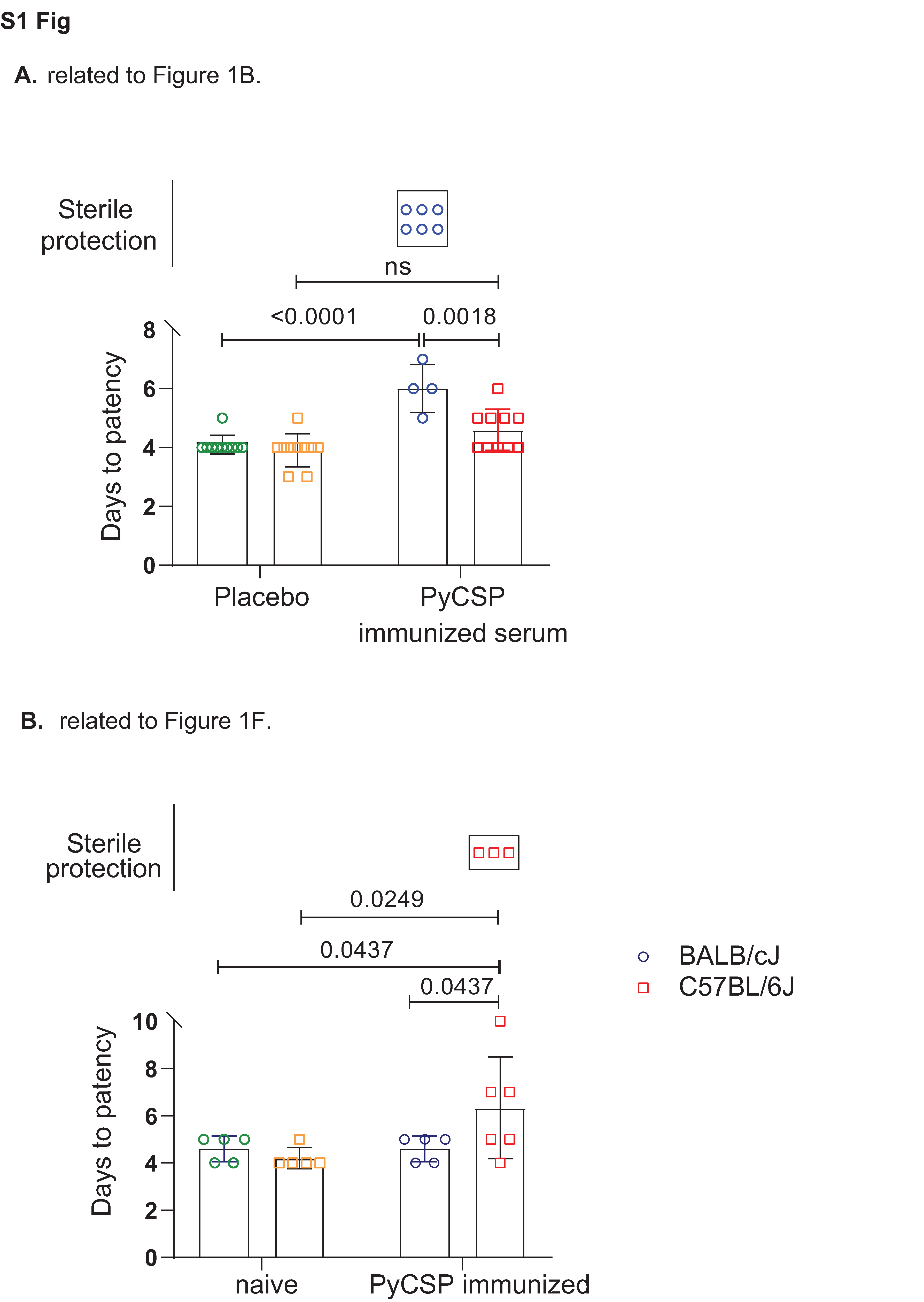

Supplement: S1 Fig — A. Graphics showing the statistical significance of PyCSP-immunized BALB/cJ (blue), C57BL/6J (red) and their respective placebo controls in green and yellow. B. Statistics showing the significance of the delay in blood stage patency in swapped PyCSP-pAbs (90 μg)-infused BALB/cJ (blue), C57BL/6J (red) and their respective placebo controls in green and yellow. The days to patency are measured and the number of mice that are sterile protected in the PyCSP-immunized BALB/cJ mice (A) and PyCSP-immunized BALB/cJ mice pAbs infused naïve C57BL/6J mice (B) were indicated. Data analyzed by Two-way ANOVA and p values were obtained by Tukey’s multiple comparison test. (TIF) [file ppat.1010671.s001.tif]

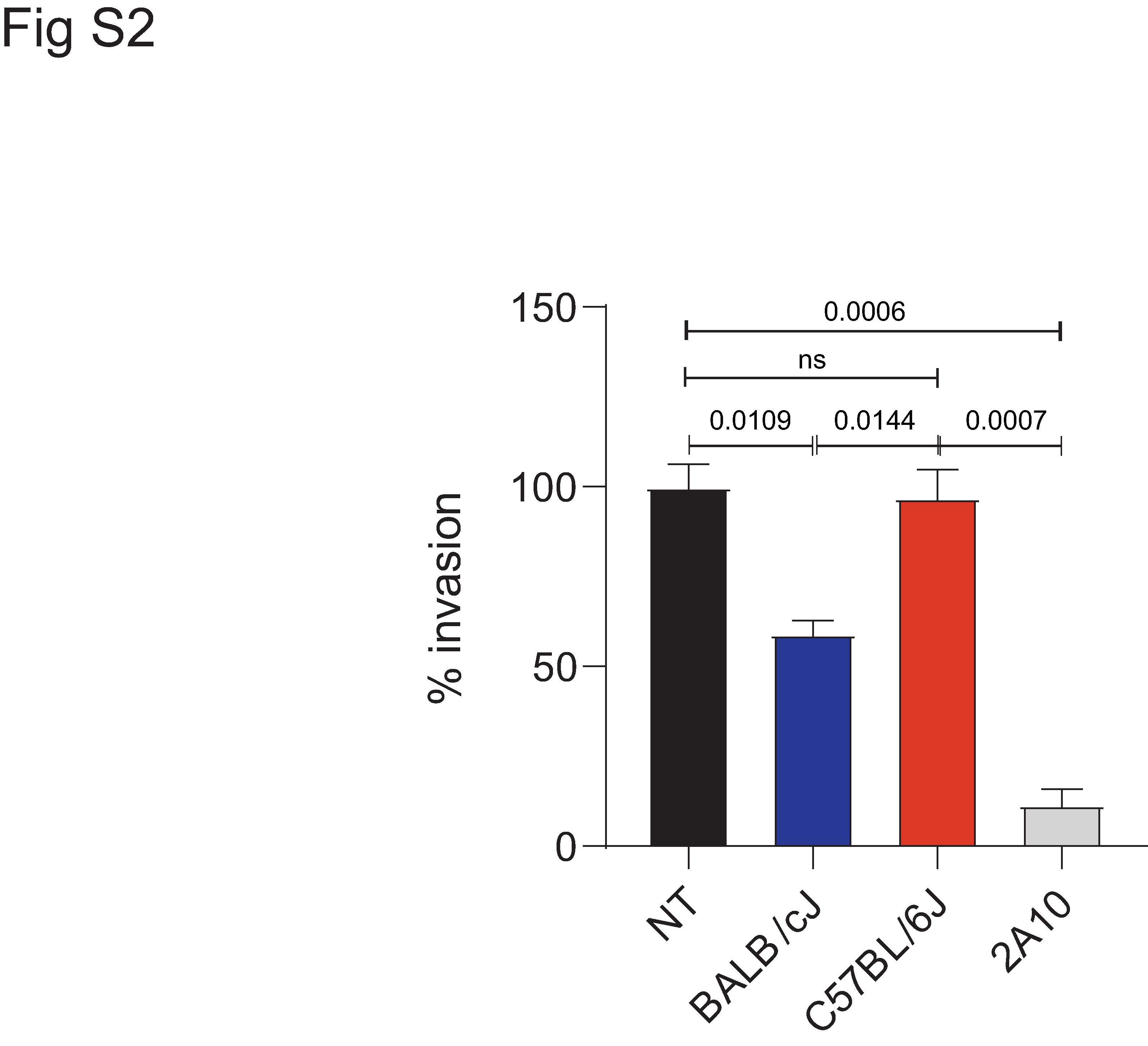

Supplement: S2 Fig — Purified pAbs (1:10) from PfCSP-immunized BALB/cJ (blue) and C57BL/6J (red) mice were assayed for in vitro functional activity as described in the Materials and Methods section. A canonical PfCSP mAb-2A10 (grey) and untreated (NT, Black) cells were used as experimental controls. (TIF) [file ppat.1010671.s002.tif]

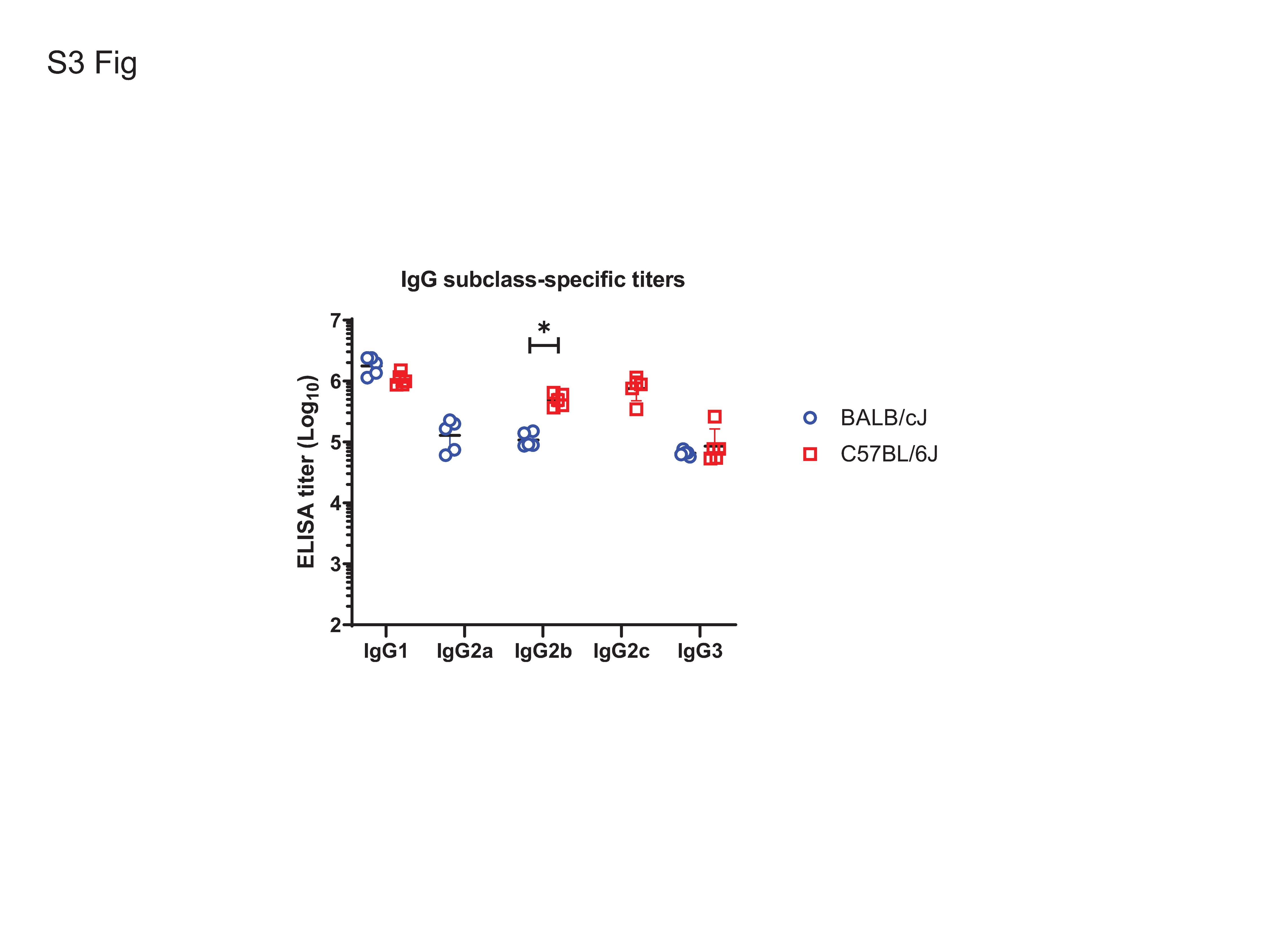

Supplement: S3 Fig — The plasma IgG1, IgG2a (only for BALB/cJ), IgG2b, IgG2c (only for C57BL/6J) and IgG3 antibody responses of PyCSP-immunized BALB/cJ (blue circles) and C57BL/6J (red squares) mice at week 7 (pre-challenge) were measured by ELISA using biotinylated PyCSP as ligand. Data analyzed by Two-way ANOVA and p values were obtained by Sidak’s multiple comparisons test. ****p<S0.0001. (TIF) [file ppat.1010671.s003.tif]

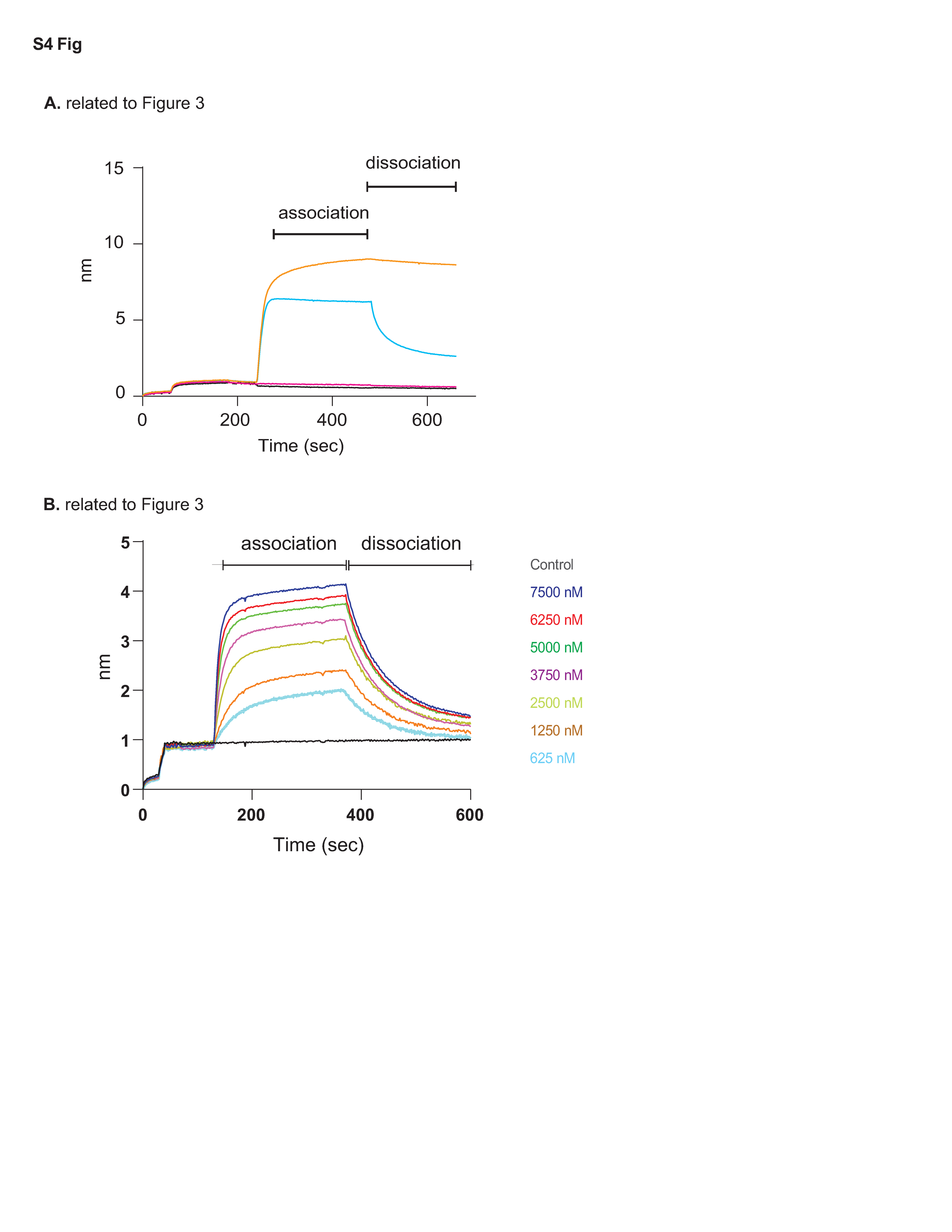

Supplement: S4 Fig — A. Biotinylated-major repeat peptide (5 μg) loaded streptavidin biosensors were dipped in 5 μg each of mAbs (RAM1-cyan, RAM2-orange, 2F6-pink and 50C1-black) and the association and dissociation kinetics were assayed by Octet-BLI. B. Streptavidin biosensors loaded with C-terminally biotinylated-PyCSP were incubated in different concentrations of RAM2 Fab ranging from 7500 nM to 625 nM and the association and dissociation kinetics were analyzed. The resulting association and dissociation sensograms were analyzed by a global fit 1:1 binding model using the ForteBio data analysis software (version 7.0.1.5) generating KD as estimated from the on- and off-rates. (TIF) [file ppat.1010671.s004.tif]

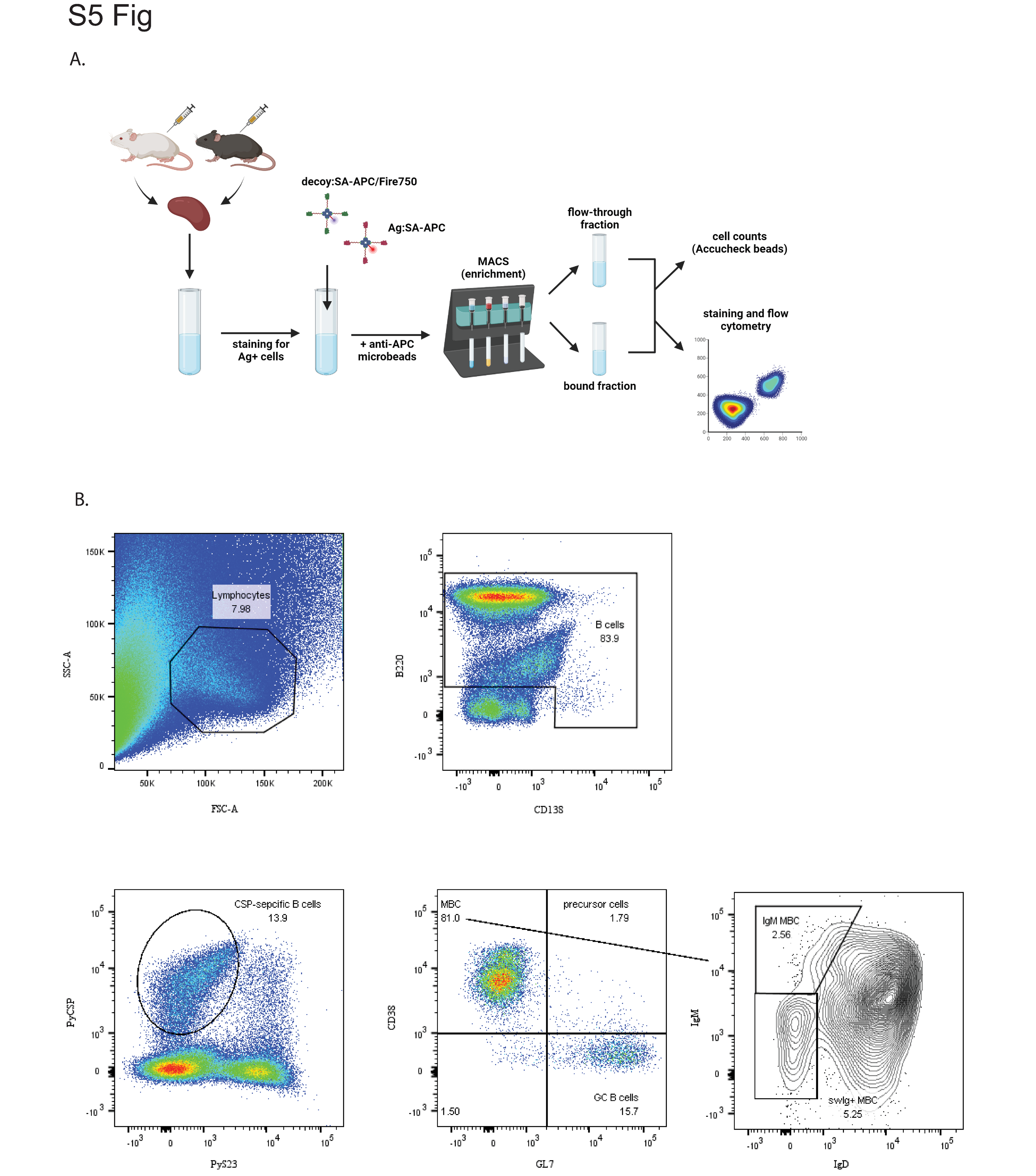

Supplement: S5 Fig — A. Summary of the workflow showing the different steps involved in the quantification of CSP-specific B-cell responses as described in the Materials and Methods section. B. Overview of the gating strategy used in Fig 4. (TIF) [file ppat.1010671.s005.tif]

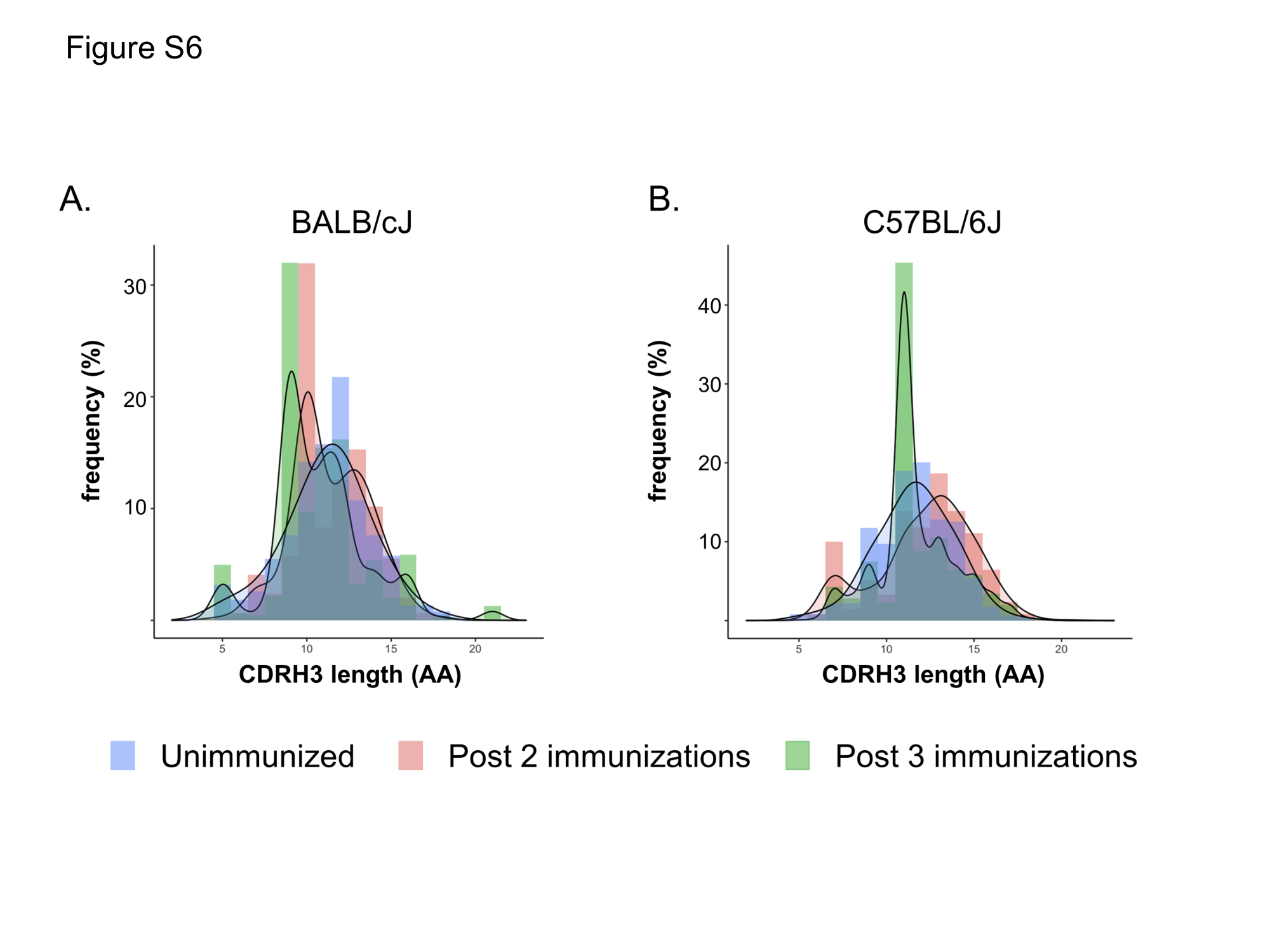

Supplement: S6 Fig — The data shown are a representative set from three independent experiments. (TIF) [file ppat.1010671.s006.tif]
