# Supplementary material for: Germinal center activity and B cell maturation are associated with protective antibody responses against Plasmodium pre-erythrocytic infection
Source: PLoS Pathog. 2022 Jul 6;18(7):e1010671. doi: 10.1371/journal.ppat.1010671 (PMC9292112; doi:10.1371/journal.ppat.1010671)
Supplement: S1 Table — (DOCX) [file ppat.1010671.s007.docx]

**S1 Table. Gene usage and CDR3 characteristics of anti-CSP mAbs**

| **mAb** | **Gene usage**  **V D J** | | | **CDR3 aa sequence** | **Reference** |
| --- | --- | --- | --- | --- | --- |
| **RAM1 –**  Heavy chain:  Light chain: | IGHV1-81*01  IGKV8-30*01 | IGHD1-1*01 | IGHJ1*01  IGKJ1*01 | ARLKYGISYEWYFDV  QQYYSYPRT | [31] |
| **RAM2 –**  Heavy chain:  Light chain: | IGHV5-9-4*01  IGKV8-30*01 | IGHD2-2*01,IGHD2-3*01,IGHD2-7*01 | IGHJ1*01  IGKJ1*01 | ARVGSDWYFDV  QQYYYYPRT | (Present study) |
| **2F6 –**  Heavy chain:  Light chain: | IGHV9-2-1*01,IGHV9-3-1*01  IGKV8-30*01 | IGHD1-1*01,IGHD1-1*02,IGHD2-4*01 | IGHJ2*03  IGKJ1*01 | ARVHYYAMDD  QQYYRLWT | [29-31] |
