# Supplementary material for: Germinal center activity and B cell maturation are associated with protective antibody responses against Plasmodium pre-erythrocytic infection
Source: PLoS Pathog. 2022 Jul 6;18(7):e1010671. doi: 10.1371/journal.ppat.1010671 (PMC9292112; doi:10.1371/journal.ppat.1010671)
Supplement: S2 Table — (DOCX) [file ppat.1010671.s008.docx]

**S2 Table. Oligonucleotides used in the NGS experiment**

| **Primer Name** | **Sequence** |
| --- | --- |
| vv-534 | TGCATTTGAACTCCTTGCC |
| vv-877 | AAGCAGUGGTAUCAACGCAGAGNNNUNNNNUNNNNUNNNNUCTTrGrGrG |
| vv-869 | AAGCAGTGGTATCAACGCAG |
| vv-870 | KKACAGTCACTGAGCTGCT |
| vv-872 | TACAGTCACCAAGCTGCT |
| vv-873 | CACTCTATCCGACAAGCAGTGGTATCAACG |
| vv-874 | GGGCCAGTGGATAGAC |
| vv-876 | GGGACCAAGGGATAGAC |
